# Interfacial Polymerization at the Alkane/Ionic Liquid Interface

**DOI:** 10.1002/anie.202103555

**Published:** 2021-05-19

**Authors:** Chang Liu, Jing Yang, Bian‐Bian Guo, Seema Agarwal, Andreas Greiner, Zhi‐Kang Xu

**Affiliations:** ^1^ MOE Key Laboratory of Macromolecular Synthesis and Functionalization Key Laboratory of Adsorption and Separation Materials & Technologies of Zhejiang Province Department of Polymer Science and Engineering Zhejiang University Hangzhou 310027 China; ^2^ College of Material Chemistry and Chemical Engineering Hangzhou Normal University Hangzhou 310036 China; ^3^ Macromolecular Chemistry and Bavarian Polymer Institute University of Bayreuth Universitatsstrasse 30 95440 Bayreuth Germany; ^4^ College of Chemical and Biological Engineering Zhejiang University Hangzhou 310027 China

**Keywords:** interfacial polymerization, ionic liquids, membranes, nanofilms, polyamides

## Abstract

Polymerization at the liquid–liquid interface has attracted much attention for synthesizing ultrathin polymer films for molecular sieving. However, it remains a major challenge to conduct this process outside the alkane–water interface since it not only suffers water‐caused side reactions but also is limited to water‐soluble monomers. Here, we report the interfacial polymerization at the alkane/ionic liquid interface (IP@AILI) where the ionic liquid acts as the universal solvent for diversified amines to synthesize task‐specific polyamide nanofilms. We propose that IP@AILI occurs when acyl chloride diffuses from the alkane into the ionic liquid instead of being triggered by the diffusion of amines as in the conventional alkane–water system, which is demonstrated by thermodynamic partitioning and kinetic monitoring. The prepared polyamide nanofilms with precisely adjustable pore sizes display unprecedented permeability and selectivity in various separation processes.

## Graphical abstract

Interfacial polymerization at the alkane/ionic liquid interface has been designed. The extendibility, designability, and controllability brought by the ionic liquid replace the instability and low solubility caused by water in conventional polymerization. The polyamide nanofilms synthesized at the new delicate water‐insoluble monomer interfaces possess adjustable pore size and exhibit unprecedented performance in various separation applications.

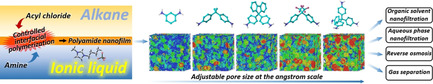

## Introduction

Liquid–liquid interfaces hold great potential for the preparation of polymeric materials since interfacial polymerization was reported by Wittbecker and Morgan in 1959.[^1^, ^2^, ^3^] This interfacial polymerization, owing to its remarkable efficiency and robustness, has evolved as a powerful platform for synthesizing functional polymers as diverse as nanofibers, capsules, and especially ultra‐thin polymer films that have been applied as separation membranes in energy‐efficient water desalination, organic solvent purification, and gas separation.[^4^, ^5^, ^6^, ^7^, ^8^, ^9^, ^10^] Generally, the conventional interfacial polymerization is a condensation reaction between amine and acyl chloride that are, respectively dissolved in water and alkane to construct polyamide nanofilms.[^2^, ^3^] However, most organic compounds including amines are difficult to be dissolved in water, especially those aromatic amines with complex topologies. Therefore, it significantly impedes the interfacial synthesis of polymeric nanofilms for task‐specific separation membranes.^[11]^ Moreover, the conventional interfacial polymerization is inevitably accompanied by a side reaction, that is, the hydrolysis of acyl chloride due to the presence of water. This side reaction leads to the existence of non‐crosslinkable sites in the synthesized polyamide nanofilms, thereby causing defects in the separation membranes and then making the practical applications to suffer a well‐known bottleneck of “trade‐off effect”.[^12^, ^13^, ^14^] It remains a major challenge to develop a water‐free interfacial polymerization with a stable interface, monomer universality, and controllable process as well as without side reactions.

**Figure 1:**
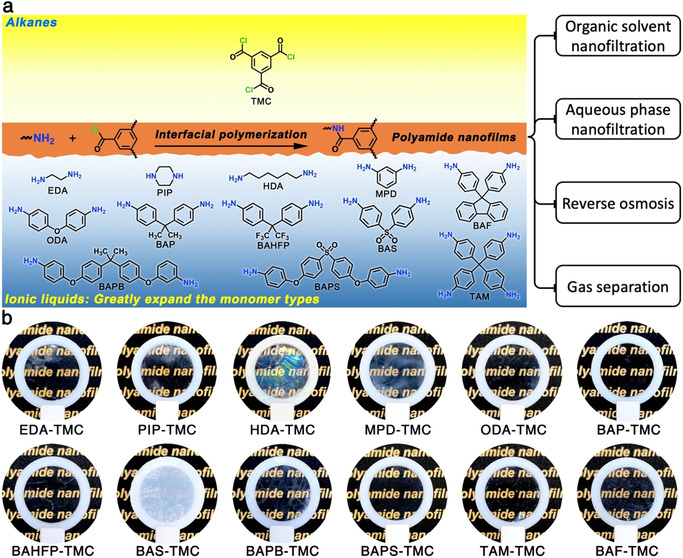
Interfacial polymerization at the alkane/ionic liquid interface for synthesizing polyamide freestanding nanofilms. a) Schematic illustration of the interface polymerization at an alkane–IL interface (IP@AILI). The amine is dissolved in IL and reacts with trimesoyl chloride (TMC) dissolved in alkane at the alkane–IL interface. Twelve amine monomers were evaluated: ethylenediamine (EDA), piperazine (PIP), hexamethylenediamine (HDA), *m*‐phenylenediamine (MPD), 4,4′‐oxydianiline (ODA), 2,2‐bis(4‐aminophenyl)propane (BAP), 2,2‐bis(4‐aminophenyl)hexafluoropropane (BAHFP), bis(4‐aminophenyl)sulfone (BAS), 1,4‐bis(4‐aminophenoxy)benzene (BAPB), bis[4‐(4‐aminophenoxy)phenyl]sulfone (BAPS), tetrakis(4‐aminophenyl)methane (TAM), 9,9‐bis(4‐aminophenyl)fluorene (BAF). These amines show different molecular structures, topographies, and electrostatic potentials (Figure S3). b) Digital photographs of the polyamide freestanding nanofilms transferred to a Teflon ring. Each sample name below refers to the monomer composition for the IP@AILI.

Ionic liquids (ILs) are a type of room‐temperature molten organic salts immensely distinguished from water. They possess a set of intriguing merits such as extremely low vapor pressure, high stability, powerful solvency, and rich designability.^[15]^ Their fascinating nature makes it possible to dissolve various aromatic amines while forming stable interfaces with alkanes.[^16^, ^17^] Such anhydrous interfaces can thoroughly avoid the hydrolysis of acyl chloride during interfacial polymerization. Herein, we report a series of alkane–IL interfaces, in which ILs act as universal solvents for diversified amine monomers, to synthesize polyamide nanofilms by the interfacial polymerization between the amine and trimesoyl chloride (TMC) (Figure 1). Various ILs were found to form sharp interfaces with alkanes such as n‐hexane, cyclohexane, and Isopar H (Figure S1 and S2). A large variety of water‐insoluble amines possessing different functional groups and topologies (Figure S3) were used to construct polyamide nanofilms with tailored pore structure and microporosity for different separation targets. Remarkably, we demonstrate that the interfacial polymerization at the alkane–IL interface (IP@AILI) occurs by TMC diffusing from alkane into IL rather than amine diffusing from water into alkane at the conventional alkane–water interface. Meanwhile, the extraordinary properties of inertness, high viscosity, and interfacial stability of ILs make the IP@AILI a controllable process, thus leading to the ultra‐thin thickness and high crosslinking degree for the synthesized polyamide nanofilms. Finally, the corresponding polyamide membranes exhibit unprecedented permeability and selectivity in various separation processes including reverse osmosis, aqueous nanofiltration, organic solvent nanofiltration, and gas separation. Our discovery offers wide perspectives for interfacial polymerization as well as the rational design of sophisticated separation membranes.

## Results and Discussion

### Properties of the Interfaces Formed by Ionic Liquids and Alkanes

**Figure 2:**
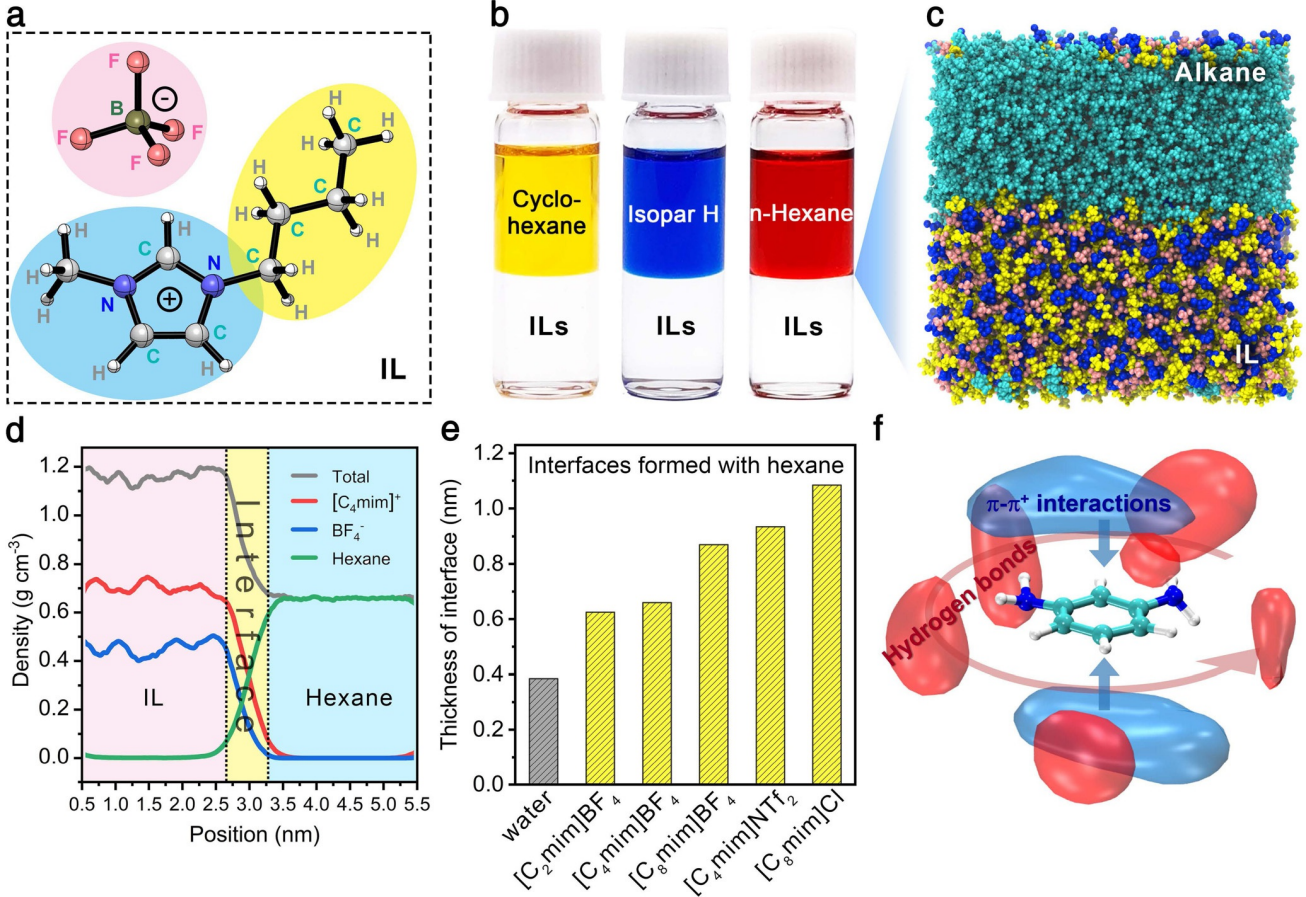
Profiles of the alkane–IL interfaces and solvency of ILs. a) Chemical structure of [C_4_mim]BF_4_, where the silver‐gray, blue, white, olive, and pink spheres represent carbon, nitrogen, hydrogen, boron, and fluorine atoms, respectively. The blue, yellow, and pink shadows represent methylimidazole, alkyl substituent, and tetrafluoroborate anion, respectively. b) Digital photograph of immiscible alkane/[C_4_mim]BF_4_ systems with a clear interface. Cyclohexane, Isopar H, and *n*‐hexane were dyed yellow, blue, and red, respectively. c) Snapshot of the hexane/[C_4_mim]BF_4_ (cyan) interface in the molecular dynamic simulation. The color code of [C_4_mim]BF_4_ is the same as in (a). Inset details the orientation of 1‐butyl‐3‐methylimidazole cation at the alkane–IL interface. d) Density distribution of [C_4_mim]BF_4_ and hexane across the alkane–IL interface. Two endpoints with 90 % of bulk density for each phase were defined as the boundary line of the alkane–IL interface. e) The thickness of the interfaces formed by *n*‐hexane with water and different ILs. f) Image of the spatial distribution function of [C_4_mim]^+^ (blue) and BF_4_^−^ (red) around the MPD molecule.

We selected various imidazolium ILs and three alkanes (n‐hexane, cyclohexane, and Isopar H) as typical examples to elucidate the chemical compositions, the interfacial structures, and the physical characteristics of the alkane–IL interfaces. A stable phase interface with a sharp boundary can be facilely formed by pouring alkane on the surface of IL for all studied cases (Figure S4). Taking 1‐butyl‐3‐methylimidazolium tetrafluoroborate ([C_4_mim]BF_4_, Figure 2 a) for instance, molecular dynamics simulations show an equilibrated interfacial structure of hexane–[C_4_mim]BF_4_ with a thickness of ≈0.64 nm (Figure 2 c and d), which is thicker than 0.40 nm of the hexane–water interface due to the large size of the alkyl imidazolium cation moiety of [C_4_mim]BF_4_. The interface thickness increases with the length of alkyl substituent in ILs (Figure 2 e and Figure S4). Note that the alkyl substituents would insert into the alkane phase at the alkane–IL interface via the van der Waals interaction,^[18]^ resulting in low interfacial tension (Figure S5 and Table S1). This low interfacial tension synergized with the high viscosity of ILs can significantly suppress the Marangoni effect at the alkane–IL interface, leading to high interfacial stability with low perturbance (Figure S6 and Supplemental Movie).[^19^, ^20^] All these characteristics offer more flexibility and dimension than the alkane–water interface in designing and controlling the interfacial polymerization between amines and acyl chlorides.

The solubility of monomers, especially amines in ILs, is a critical issue for the interfacial polymerization of amine with acyl chloride. We investigated it from the molecular‐level perspectives by simulating the spatial distribution function of cation and anion around an amine molecule in IL solutions. Taking MPD as a typical example, the result clearly shows that the hydrogen bonds and the π‐π^+^ interactions promote the good dissolution of amines in ILs (Figure 2 f).^[21]^ It is worth noting that although ILs are not fully inert, they are still stable solvents in IP@AILI (Figure S7 and Table S2).[^22^, ^23^, ^24^] These facts indicate that even those hydrophobic amines with aromatic rings (Figure 1 a and Figure S3) can be safely and smoothly dissolved in ILs, thus providing prerequisites for the synthesis of task‐specific polyamide nanofilms by the IP@AILI.

### Interfacial Polymerization Mechanism at the Alkane/Ionic Liquid Interface

**Figure 3:**
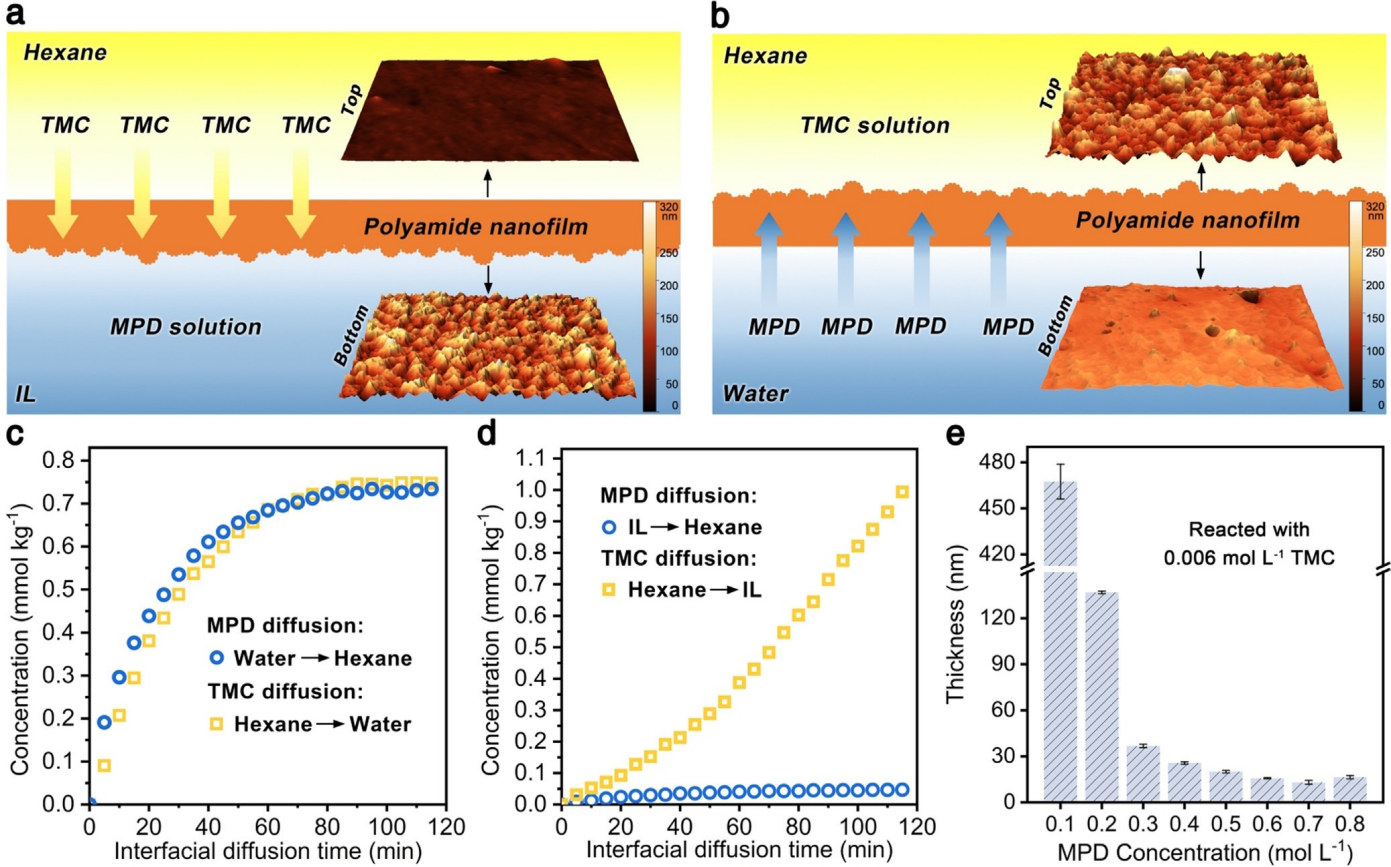
Characteristics of the IP@AILI. a,b) Schematic illustrations of the monomer diffusion direction in a) the IP@AILI and b) the conventional interfacial polymerization at the alkane–water interface. The insets show the surface morphologies of the resulting polyamide nanofilms visualized by AFM. c,d) Monomer concentration detected at a position 30 μm away from c) the alkane–water interface and d) the alkane–IL interface via UV spectroscopy versus interfacial diffusion time. e) The thickness of polyamide nanofilm measured by ellipsometry versus MPD concentration (reacted with 0.56 mm TMC, 10 min).

We further explored the diffusion mechanism of acyl chloride and amine monomer at the alkane–IL interfaces, which is also critical to the synthesis of polyamide nanofilms. In conventional interfacial polymerization, it has been widely accepted that amine monomer in the aqueous phase diffuse across the alkane–water interface and then react with acyl chlorides in the alkane phase.^[11]^ This diffusion mechanism is ascribed to the higher solubility of amine in alkane than that of acyl chloride in water.^[12]^ To inspect the monomer diffusion mechanism across the alkane–IL interface, we calculated the partition coefficient (log *P*_A/B_) of MPD and TMC between immiscible solvents A (n‐hexane) and B (water or IL) according to the principle of chemical equilibrium [Eq. 1]:

(1) $${\log P}_{A/B}=\log\frac{c_{A}}{c_{B}}=-\frac{\Delta G_{solv}\left(A\right)-\Delta G_{solv}\left(B\right)}{2.303RT}$$

where *c* and Δ*G*_solv_ are the concentration and the free energy of the solute in the solvent, *R* is the ideal gas constant (8.314 J K mol^−1^), and *T* is the absolute temperature.^[25]^ Density functional theory calculations show that the log *P* value of MPD in the cases of hexane–water and hexane–[C_4_mim]BF_4_ is −2.8 and −3.6, respectively (Table S3). These results suggest that MPD is more difficult to diffuse into the hexane phase when [C_4_mim]BF_4_ was used as a solvent compared with water. Meanwhile, the log *P* value of TMC in the cases of hexane–water and hexane–[C_4_mim]BF_4_ is 4.4 and 0.9, respectively, indicating that TMC is more willing to diffuse from hexane into [C_4_mim]BF_4_ rather than water. Considering these results, we propose an alternative diffusion‐reaction model describing the IP@AILI. That is, the polymerization occurs when acyl chloride diffuses from the alkane phase into the IL phase and reacts with amine monomers (Figure 3 a). This model describes the opposite diffusion direction of monomers compared with the conventional interfacial polymerization at the alkane–water interface (Figure 3 b).

It can be further manifested by tracking the diffusion kinetics of monomers across the interfaces in real‐time with UV spectroscopy (Figure S8 and S9). Both MPD and TMC undergo a rapid diffusion at the hexane–water interface. The equilibrium concentrations are reached after ≈90 min in the absence of polymerization (Figure 3 c). Considering that TMC is almost insoluble in water, the diffusion detected actually comes from those hydrolyzed species (carboxylic acid). In vivid contrast, the two monomers exhibit slow diffusion rates in the alkane–IL interface at the primary stage (Figure 3 d), making the interfacial polymerization more moderate and controllable, and thus the resulting polyamide nanofilms are thinner and smoother than the conventional one (Figure S10 and S11). Notably, the diffusion rate of TMC from hexane to IL is much higher than that of MPD from IL to hexane, indicating that the polyamide nanofilms gradually grow along the same direction of TMC diffusion in the IL phase rather than in the alkane phase. Therefore, the polyamide nanofilms possess smooth top surfaces (hexane‐facing) while crumpled lower surfaces (IL‐facing), which is opposite to the topography of those synthesized at the hexane–water interface (Figure 3 a and b insets, and Figure S10). After polymerization, the by‐product hydrogen chloride is bound by excess amine to form ammonium salt and dissolve in IL (Figure S12 and S13). Moreover, due to an antagonistic relationship between polymerization and diffusion so‐called self‐limiting effect, when the concentration of TMC is fixed, the higher the local concentration of amine, the faster the polymerization rate and the earlier the formation of a dense film, which will inhibit the further diffusion of TMC and fix the thickness of synthesized polyamide nanofilm on a low value. Thus, the amine can be regarded as a terminator for the diffusion of acyl chloride (Figure 3 e and Figure S14). All these characteristics endow the polyamide nanofilms with the superiority of controllable thickness.

### Highly Crosslinked Polyamide Nanofilms for Aqueous Nanofiltration and Reverse Osmosis

**Figure 4:**
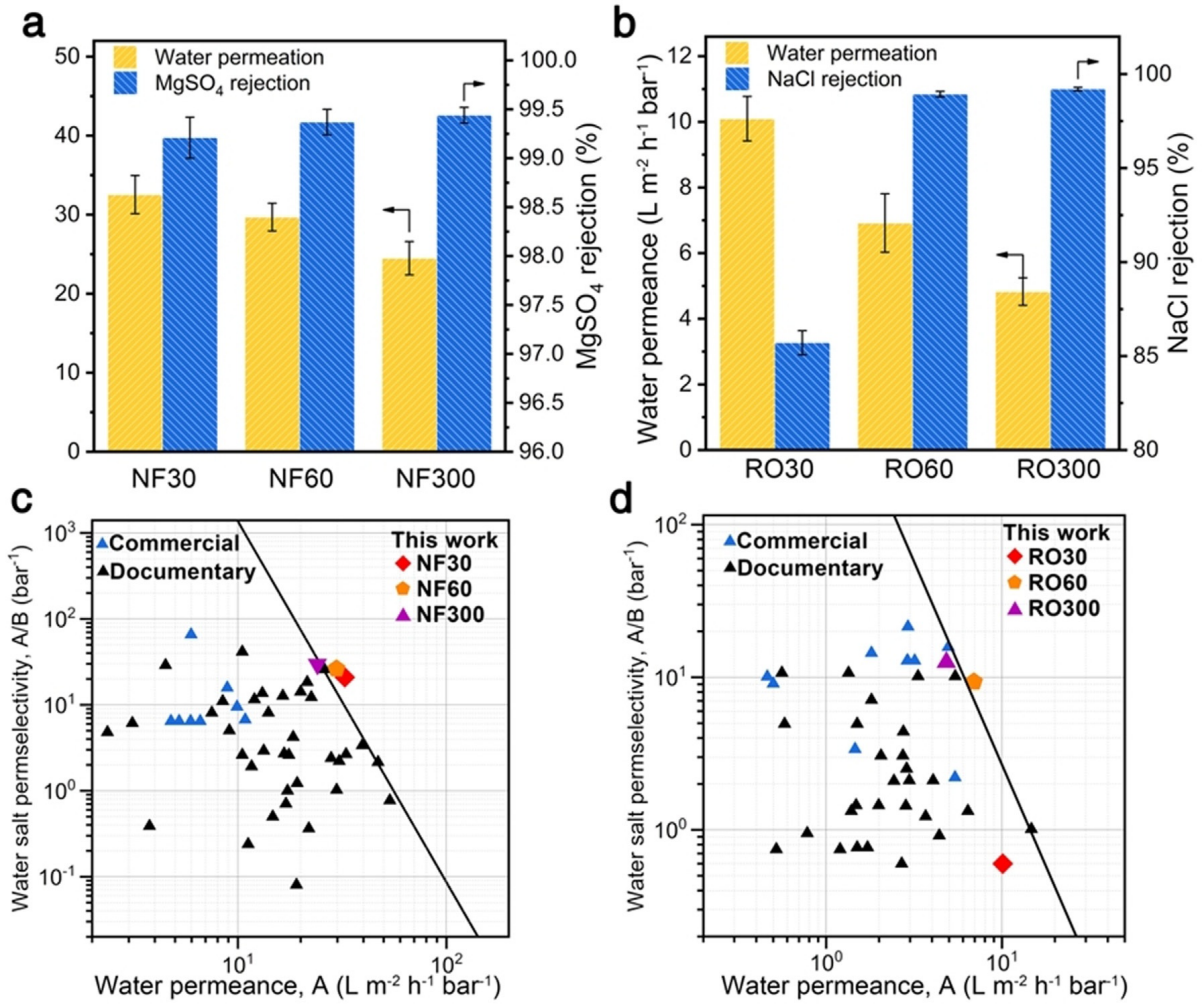
Nanofiltration and reverse osmosis properties of the polyamide‐based membranes synthesized by the IP@AILI. a,b) Water permeation and salt rejection of the polyamide‐based membranes for a) nanofiltration and b) reverse osmosis. The nanofiltration membranes named NF30, NF60, and NF300 were prepared by the IP@AILI of PIP and TMC for 30, 60, and 300 s which have a thickness of 6.5 nm, 12 nm, and 23 nm, respectively. The reverse osmosis membranes named RO30, RO60, and RO300 were prepared by the IP@AILI of MPD and TMC for 30, 60, and 300 s which have a thickness of 2.3 nm, 10.2 nm, and 12.5 nm, respectively (120 mm PIP or 500 mm MPD reacted with 0.56 mm TMC). Aqueous solutions of MgSO_4_ (1000 ppm) and NaCl (2000 ppm) were used as the feed solutions for nanofiltration and reverse osmosis, respectively. c,d) Correlation between the water–salt permeaselectivity and the water permeance for the polyamide‐based membranes in c) nanofiltration and d) reverse osmosis. Blue and gray triangles represent commercial and documentary membranes, respectively. The solid black line is the upper‐bound line manually drawn based on the data points which can be found in Table S4 and Table S5 in the Supporting Information, respectively.

It is noteworthy that the hydrolysis of acyl chloride at the alkane–water interface is an inevitable issue for the conventional interfacial polymerization because the huge number of water molecules is dominant even if the reactivity of amine (9500 mol^−1^ L^−1^ s^−1^ of the rate constant) is higher than that of water (1500 mol^−1^ L^−1^ s^−1^ of the rate constant).^[26]^ This hydrolysis reduces the crosslinking degree and increases the negative charges of the polyamide nanofilms (Figure S15), which makes the alkane–water interface challenging to synthesize ultrathin and defect‐free nanofilms for alleviating the bottleneck of permeability‐selectivity trade‐off in desalination. Our IP@AILI has the advantage to prepare defect‐free polyamide nanofilms with a high crosslinking degree because the hydrolysis side reaction can be avoided at the anhydrous interface. To manifest this conjecture, polyamide nanofilms with a thickness of sub‐20 nm were synthesized from MPD and PIP with TMC, respectively, at the hexane–[C_4_mim]BF_4_ interface. These nanofilms were then deposited on the anodic aluminum oxide discs for evaluating their separation performances (Figure S16–S19) by nanofiltration and reverse osmosis. They show excellent permeability and selectivity (Figure 4 a and b), exceeding the water‐salt separation upper bound line of commercial and documentary membranes to date (Figure 4 c and d). This performance is mainly ascribed to the ultra‐low thickness and the high crosslinking degree of the polyamide nanofilms prepared by the IP@AILI (Figure S20, S21, and Table S6). Meanwhile, these results are in contrast to the “trade‐off effect” of the conventional membranes whereby high water permeability invariably leads to low salt rejection. Note that the RO30 membrane shows a low salt rejection (≈85 %) (Figure 4 b and Figure S19) because the polyamide nanofilm is too thin for desirable performance (2.3 nm).

### Pore‐Tailored Polyamide Nanofilms for Organic Solvent Nanofiltration and Gas Separation

**Figure 5:**
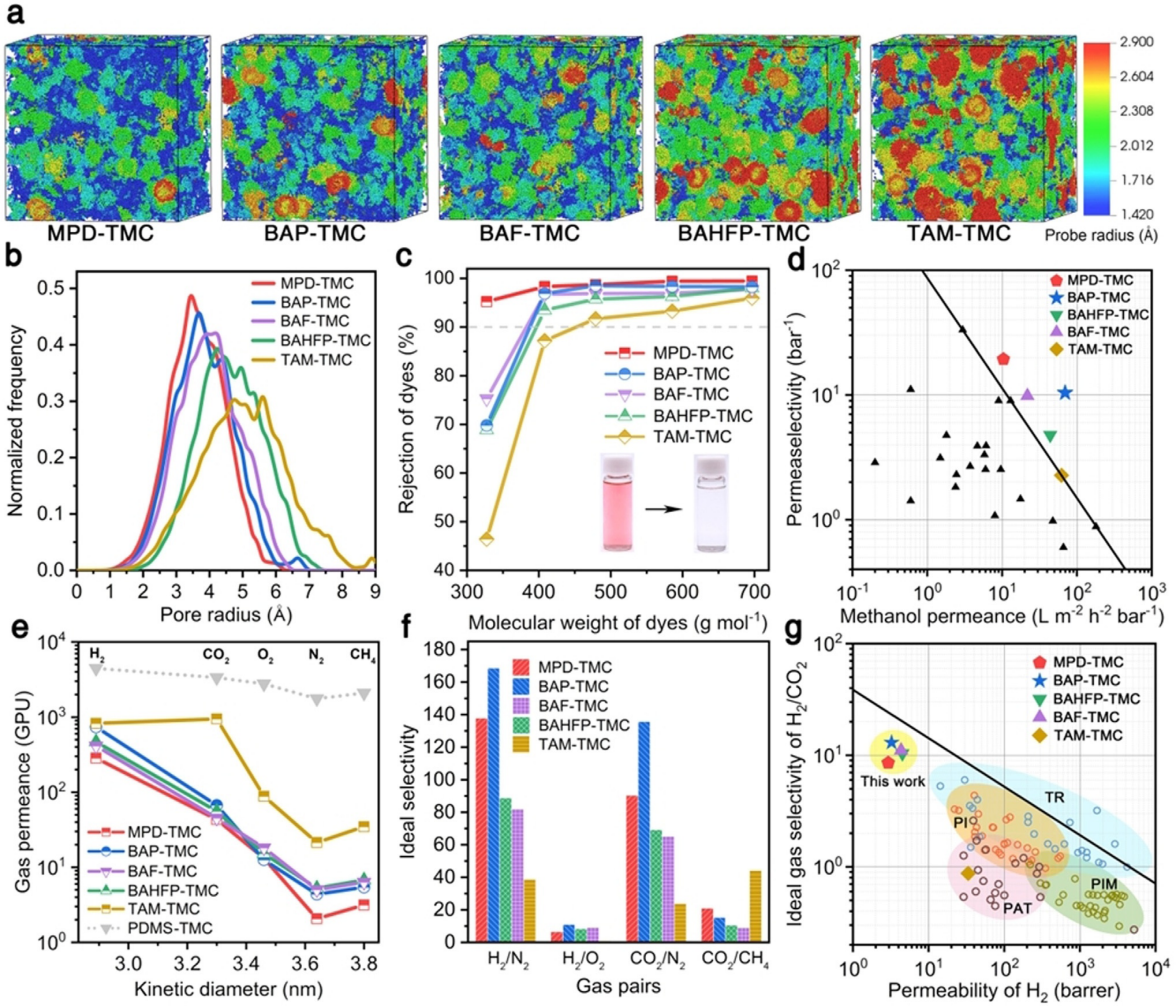
The microporous structure of polyamide membranes synthesized by IP@AILI and their performances in organic solvent nanofiltration and gas separation. a) Coloring diagrams concerning the micropore radius of polyamide nanofilms synthesized by different monomer combinations. b) Simulated pore size distributions of polyamide nanofilms. c) Rejection versus the molecular weight of dyes in methanol (methyl orange, 327.3 g mol^−1^; gentian violet, 408.0 g mol^−1^; rhodamine B, 479.0 g mol^−1^; acid fuchsin, 585.5 g mol^−1^; Congo red, 696.7 g mol^−1^). Inset photos show the retentate (left) and permeate (right) solution. d) The permeance of methanol versus selectivity toward small solutes (320–410 g mol^−1^) for thin‐film composite membranes prepared by IP@AILI in this work and those documentary ones synthesized on the alkane–water interface. The upper‐bound line is manually added to show a trade‐off between solvent permeance and permeaselectivity. The data of points can be found in Table S9 in the Supporting Information. e) Gas permeance as a function of the kinetic diameter of gas molecules through microporous polyamide nanofilm mounted on PDMS thin films (50 nm) spin‐coated on an anodic aluminum oxide substrate. Gas permeation unit (GPU): 1 GPU=10^−6^ cm^3^ (STP) cm^−2^ s^−1^ cmHg^−1^. f) Ideal gas selectivity for typical gas pairs. g) The upper‐bound plot of H_2_/CO_2_ selectivity versus permeability of H_2_ for microporous polyamide‐based thin‐film composite membranes. Permeability data were calculated based on the thickness of the polyamide layer. The upper bound line of polymeric membranes was plotted (see ref. [26]). Typical high‐free‐volume PIM polymers (olive circles), thermal rearrangement polymer (TR, blue circles), polyacetylene (PAT, brown circles), and polyimide (PI, orange circles) were included. The data of points can be found in Table S10.

Additionally, the IP@AILI offers more freedom to tailor the structures and properties of the polyamide nanofilms owing to the universal dissolving ability of ILs for many amine monomers. Those contorted aromatic amines, which are insoluble in water and thus unusable in the conventional interfacial polymerization, are especially potent for enhancing the microporosity and interconnectivity of polyamide networks. The as‐formed rigid and non‐coplanar polymer backbones are insufficiently packed to form interconnected voids of less than 2 nm that behave as micropores.[^7^, ^27^] Our IP@AILI provides great possibilities to synthesize various polyamide nanofilms with tunable microstructure by simply selecting amines possessing different structures (Figures 1 a and b). We invoked computer simulations to delicately screen amine monomers for designing specific pore structures of polyamide networks. TMC and five amines (MPD, BAP, BAHFP, BAF, and TAM) were chosen for constructing polyamide nanofilms with tunable pore size. The rigid and contorted structure of amine is responsible for increasing the inherent microporosity (Figure 5 a and Table S7) of the crosslinked polyamide networks compared to the planar amine, MPD. Meanwhile, the pore size shows continuous adjustment in an angstrom‐scale (Figure 5 b), which is also confirmed by X‐ray scattering spectroscopy (Figure S22). Both free volume and pore size follow an order of MPD‐TMC < BAP‐TMC < BAF‐TMC < BAHFP‐TMC < TAM‐TMC. TAM can produce a tetrahedral crosslinking polyamide network with TMC. This stretched topology exhibits increased free volume to form large‐sized voids compared with those synthesized by difunctional amines (BAP, BAF, and BAHFP).^[28]^ With the same topological orientation, the steric hindrance induced by large‐volume CF_3_ groups leads to insufficient stacking of polyamide chains with increased pore size, i.e., BAP < BAHFP. Interestingly, although the huge fluorenyl cardo group leads to the expansion of the spatial topology, it also fills the voids between the polyamide chains, resulting in a relatively small micropore volume of BAF‐TMC (Figure S23 and Table S8).

The enhanced microporosity and the superior tunability of pore size at the angstrom‐scale endow the polyamide nanofilms with rapid and selective mass transport performances. We prepared thin‐film composite membranes containing the aforementioned polyamide nanofilms as the separation layers with thickness ranging from 4.4 to 40 nm (Figure S24). Their organic solvent permeances are in agreement with the phenomenological transport model (Figure S25).^[29]^ Specifically, the composite membranes display extremely high permeances to methanol and acetonitrile with relatively low molar volume, low viscosity, and high polarity. Meanwhile, all membranes show generally low permeance to hexane because of the non‐polar nature despite a slight lift achieved by BAHFP‐TMC on which trifluoromethyl groups can reduce the surface energy of polyamide nanofilm (Figure S26).^[9]^ Considering the compatibility of solubility parameters, the hydrophobicity of polyamide nanofilms (Figure S27) stemmed from water‐insoluble amines makes the composite membranes more suitable for organic solvent nanofiltration. For a given organic solvent, the permeance basically conforms to a sequence of MPD‐TMC < BAHFP‐TMC < TAM‐TMC < BAF‐TMC, where the high permeance for BAP‐TMC is mainly attributed to the ultrathin polyamide nanofilm with a thickness of 4.4 nm (Figure S24b). Furthermore, the rejection of solutes with different molecular weights in methanol indicates that the molecular weight cut‐off increases with the pore size (Figure 5 c and Figure S28). Figure 5 d demonstrates the correlation between methanol permeance and permeaselectivity of our membranes synthesized by IP@AILI. In contrast to other counterparts, our composite membranes with a hydrophobic surface and tunable microporosity display excellent organic solvent nanofiltration performance, surpassing the separation upper‐bound line of most reported results.

These tunable microporous structures also make our polyamide nanofilms benefit for gas separation. Figure 5 e illustrates that TAM‐TMC has the highest gas permeability despite its relatively thick polyamide nanofilm (≈40 nm, Figure S24e), manifesting the advantage of large micropore size and high free volume for rapid gas transport. Except for TAM‐TMC, the gas permeability for other thin‐film composite membranes decreases with increasing the kinetic diameter of gas (H_2_ > CO_2_ > O_2_ > N_2_ ≈ CH_4_), indicating a typical molecular sieving mechanism. All membranes exhibit much lower permeances to large‐sized gas molecules, like N_2_, leading to much higher selectivity for H_2_/N_2_. While for those gas couples with a close kinetic diameter (H_2_/O_2_), the composite membranes show low selectivity (Figure 5 f). The correlation of H_2_ permeance versus H_2_/CO_2_ selectivity demonstrates that our membranes exhibit higher selectivity than reported ones despite reduced gas permeance (Figure 5 g).^[30]^

## Conclusion

Overall, we have designed the alkane–IL interfaces with high stability and tunable interfacial thickness to provide a universal reaction location for the interfacial polymerization of various amine monomers with acyl chloride. We have revealed that interfacial polymerization occurs when acyl chloride diffuses across the interface and reacts with the amine in the IL phase, which is immensely distinct from the conventional interfacial polymerization at the alkane–water interface. The as‐synthesized polyamide nanofilms are defect‐free because the side reaction of acyl chloride can be avoided in the IP@AILI. Owing to the universal solubility of IL toward a wide range of amine monomers, the polyamide nanofilms can be finely tailored for microporous structures, offering outstanding performances in aqueous nanofiltration, reverse osmosis, organic solvent nanofiltration, and gas separation. From a broader perspective, our work provides a wide platform for interfacial polymerization to design and prepare advanced membranes. Notably, the IP@AILI can be easily implemented for the commercially scalable fabrication of task‐specific polyamide membranes with minimal alterations of technical process and manufacturing equipment. It also holds great potential for the synthesis of other cross‐linked nanofilms such as polyesters, polyurethanes, and polyuria, which are of great interest for academic researches and industrial applications. This unique interfacial polymerization approach can be also considered for other polymer structures like fibers and particles for various potentials. Surely, it could be also considered for interfacial polymerizations which were not possible with the established systems and thereby open promising avenues to advanced polymer systems.

## Conflict of interest

The authors declare no conflict of interest.

## Supporting information

As a service to our authors and readers, this journal provides supporting information supplied by the authors. Such materials are peer reviewed and may be re‐organized for online delivery, but are not copy‐edited or typeset. Technical support issues arising from supporting information (other than missing files) should be addressed to the authors.

Supplementary

Supplementary
