# Supplementary material for: Interfacial Polymerization at the Alkane/Ionic Liquid Interface
Source: Angew Chem Int Ed Engl. 2021 May 19;60(26):14636–43. doi: 10.1002/anie.202103555 (PMC8252436; doi:10.1002/anie.202103555)
Supplement: Supplementary file 1 — Supplementary [file ANIE-60-14636-s001.pdf]

Angewandte Chemie
Eine Zeitschrift der Gesellschaft Deutscher Chemiker

Supporting Information

Interfacial Polymerization at the Alkane/Ionic Liquid Interface

Chang Liu[+], Jing Yang[+], Bian-Bian Guo, Seema Agarwal, Andreas Greiner, and Zhi-Kang Xu**

anie_202103555_sm_miscellaneous_information.pdf
anie_202103555_sm_Movie.mp4

Table of Contents

Experimental Procedures 2
1. Chemicals and Materials 2
2. Methods 3
2.1. Characterization methods 3
2.1.1. Scanning electron microscopy 3
2.1.2. Transmission electron microscopy 3
2.1.3. Atomic force microscopy 3
2.1.4. Contact angle and interfacial tension measurements 3
2.1.5. Spectroscopic ellipsometry 3
2.1.6. X-ray photoelectron spectrometer 4
2.1.7. Zeta potential measurement 4
2.1.8. Wide-angle X-ray scattering 4
2.2. Experimental methods 4
2.2.1. Visualization of the alkane-ionic liquid interface 4
2.2.2. Calculation of the electrostatic potential distribution of amine monomers 6
2.2.3. Simulation of the alkane-IL interface to determine the interfacial thickness and tension 7
2.2.4. Spatial distribution function of $[C_4mim]^+$ BF_4^- around amine monomers 10
2.2.5. Visualization of interfacial turbulence 10
2.2.6. Discussion on the chemical stability of ILs 11
2.2.7. Calculation of monomer partition coefficients in different solvent systems 12
2.2.8. In situ monitoring of interfacial diffusion by UV spectroscopy 13
2.2.9 Surface morphology, chargeability, and thickness of the resulting nanofilms 16
2.2.10. Fabrication of thin-film composite membranes used for liquid phase separations 19
2.2.11. Aqueous nanofiltration and reverse osmosis 21
2.2.12. Elemental composition and crosslinking degree of the polyamide nanofilms 25
2.2.13. Structure information of the crosslinked polyamide networks 28
2.2.14. Organic solvent nanofiltration 30
2.2.15. Fabrication of freestanding polyamide nanofilms and gas separation membranes 34
2.2.16. Gas separation 36
References 39
Author Contributions 43

Experimental Procedures

1. Chemicals and Materials

Ionic liquids, including 1-butyl-3-methylimidazolium tetrafluoroborate ([C_4mim]BF_4), 1-hexyl-3-methylimidazolium tetrafluoroborate ([C_6mim]BF_4), 1-octyl-3-methyl- imidazolium tetrafluoroborate ([C_8mim]BF_4) 1-hexyl-3-methylimidazolium chloride ([C_6mim]Cl), 1-octy-3-methylimidazolium chloride ([C_8mim]Cl), 1-decyl-3-methyl- imidazolium chloride ([C_{10}mim]Cl), and 1-ethyl-3-methylimidazolium bis(trifluoromethyl- sulfonyl)imide ([C_2mim]NTf_2), were purchased from Shanghai Dibai Biotechnology, China. Piperazine (PIP) was purchased from Sigma Aldrich, US. Amines, including ethylenediamine (EDA), hexamethylenediamine (HDA), m-phenylenediamine (MPD), p-phenylenediamine (PPD), 4,4'-oxydianiline (ODA), 2,2-bis(4-aminophenyl)propane (BAP), 2,2-bis(4-aminophenyl)-hexafluoropropane (BAHFP), 4,4'-diaminodiphenylsulfone (DDS), bis(4-aminophenyl)-sulfone, 1,4-bis(4-aminophenoxy)benzene (BAPB), 2,2-bis[4-(4-aminophenoxy)phenyl]-propane (BAP), bis[4-(4-aminophenoxy)phenyl] sulfone (BAPS), 9,9-bis(4-aminophenyl)-fluorene (BAF), and tetrakis(4-aminophenyl)- methane (TAM), were bought from Aladdin Industrial, China. Trimesoyl chloride (TMC, >99%) was supplied by Qingdao Benzo Chemical, China. Anodic aluminum oxide (AAO) substrates (60 μm thick, with a mean pore size of 20 nm and diameters of 25mm and 47 mm, were purchased from Whatman, UK. Methanol, ethanol, isopropanol, acetonitrile, dimethyl sulfoxide (DMSO), *N,N*-dimethylformamide (DMF), *n*-hexane, and cyclohexane were bought from Sinopharm Chemical Reagent, China. Isopar H™ was obtained from Exxon Mobil, US. Oil red O, solvent blue 36, solvent yellow 56, congo red, acid fuchsin, rhodamine B, gentian violet, and methyl orange were purchased from Aladdin Industrial, China. Polyethylene glycol (PEG) with nominal molecular weight of 200 g mol^{-1}, 400 g mol^{-1}, 600 g mol^{-1} 800 g mol^{-1}, and 1000 g mol^{-1} was obtained from Aladdin Industrial, China. Polydimethylsiloxane (PDMS) was obtained from Shandong Dayi Chemical, China. Tetraethoxysilane (TEOS) and dibutyltin dilaurate (DBTL) were purchased from Aladdin Industrial, China. All reagents used as received without further purification. Experimental water was deionized and ultra-filtrated to 18.2 MΩ by an ELGA LabWater system, France.

2. Methods

2.1. Characterization methods

2.1.1. Scanning electron microscopy

Surface and cross-sectional morphologies of the nanofilms and composite membranes were observed by a field emission scanning electron microscopy (FESEM, S4800, Hitachi, Japan) with an accelerating voltage of 10 kV. All the samples were sputtered with platinum particles with a radius of ~5 nm before observation.

2.1.2. Transmission electron microscopy

The difference in convex structure and thickness of the polyamide nanofilms with monomer concentration was observed by a transmission electron microscopy (TEM, JEM-1230, Japan) at 80 kV in bright-field mode.

2.1.3. Atomic force microscopy

The polyamide nanofilms synthesized at the interface of hexane-[C_4mim]BF_4 were floated on the water surface, picked up with clean silicon wafers (10×10 mm), rinsed with ethanol, and then dried. Wafers loaded with the polyamide nanofilms were immersed in liquid nitrogen for 1 min, and then a scalpel was used to make scratches on the nanofilms. Atomic force microscopy (AFM, MultiMode, Vecco) was used to scan the scratches in tapping mode to determine the thickness of the polyamide nanofilms by the step height.

2.1.4. Contact angle and interfacial tension measurements

Water contact angle (WCA) and interfacial tension were determined with a DropMeter A-200 contact angle system (MAIST VisionInspection & Measurement Co. Ltd., China) at room temperature.

2.1.5. Spectroscopic ellipsometry

The thickness of the polyamide nanofilms loaded on silicon wafers was also measured by variable-angle spectroscopic ellipsometry (VASE, MD-2000I, J. A. Woollam, US). The spectra were collected

before and after nanofilm loading at incident angles of 60° and 70° in the wavelength range of 192-1688 nm. The results were analyzed by the Complete EASE analysis software using a multilayer model of a B-Spline layer on a silicon substrate with a natural oxide layer (1 nm). Then, the nanofilm thickness was fitted by the B-Spline method, including fitted thickness non-uniformity and bandwidth. Triplicate measurements were conducted for each sample in dry air at room temperature, and the average value was reported.

2.1.6. X-ray photoelectron spectrometer

Details in atomic components of polyamide nanofilms were analyzed by an X-ray photoelectron spectrometer (XPS, Escalab250Xi, ThermoFisher, USA) using Al Kα excitation radiation (1486.6 eV). The whole spectra were collected, ranging from 0 to 1310 eV with a ~10-nm survey depth.

2.1.7. Zeta potential measurement

A streaming potential method was used to detect the charging properties of the nanofilm and membrane surfaces. An electrokinetic analyzer (SurPASS Anton Paar, GmbH, Graz, Austria) with 1 mM KCl aqueous solution as the electrolyte solution was used to measure the zeta potential of the sample surface.

2.1.8. Wide-angle X-ray scattering

Wide-angle X-ray scattering was carried out by an X'Pert Powder diffractometer (PANalytical B.V., the Netherlands) with Cu Kα excitation radiation (λ = 1.5406 Å), using a step of 0.02° (2θ) per second.

2.2. Experimental methods

2.2.1. Visualization of the alkane-ionic liquid interface

Two milliliters of [C_4mim]BF_4, [C_6mim]BF_4, [C_8mim]BF_4, [C_6mim]Cl, [C_8mim]Cl, [C_{10}mim]Cl and [C_2mim]NTf_2 were used to form interfaces with 2 mL of n-hexane, Isopar H^{TM} and cyclohexane. The organic solvents were dyed with oil red O, solvent blue 36, and solvent yellow 56, respectively.

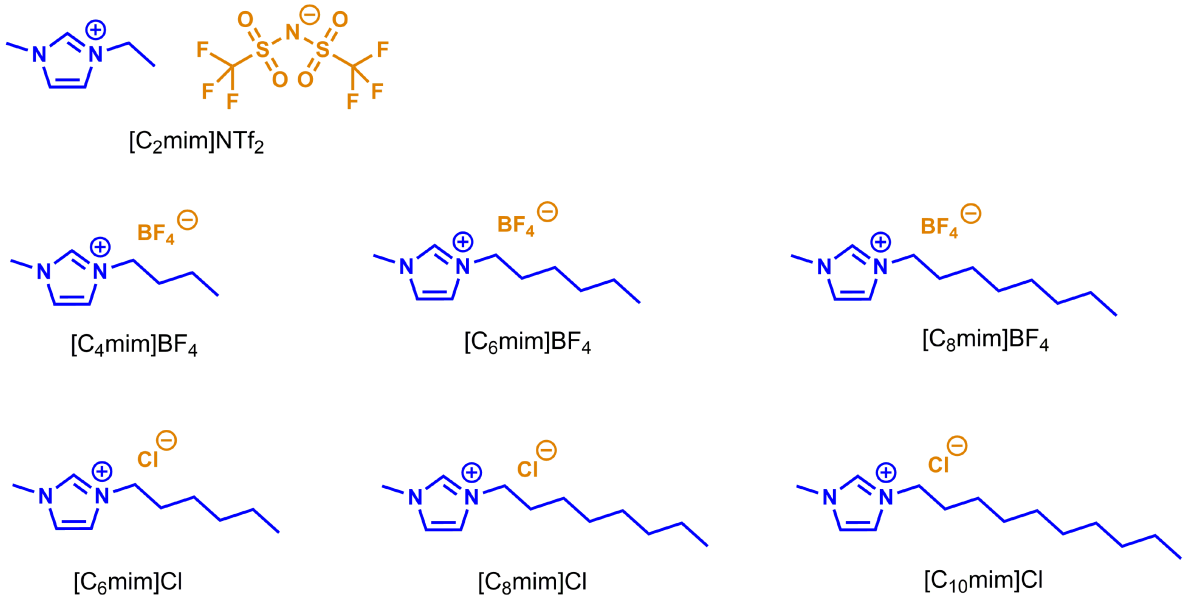

Figure S1. Chemical structures of the ILs used in this study.

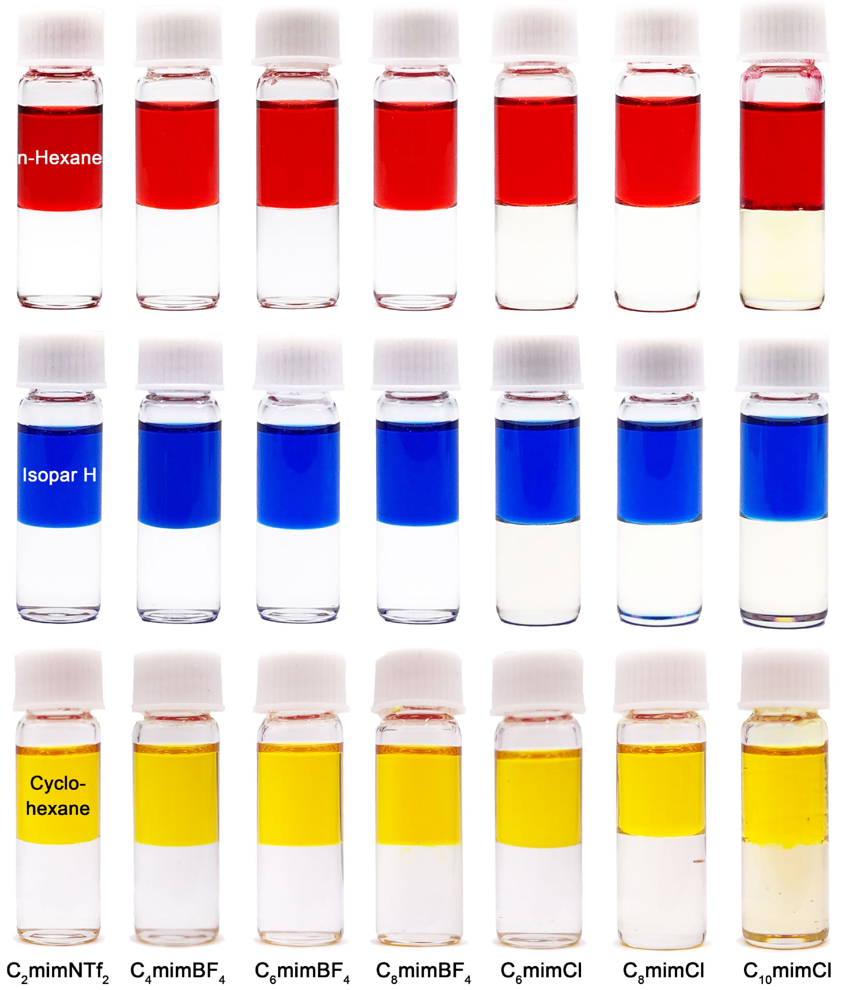

Figure S2. Photographs of the interfaces between an IL and an alkane including n-hexane (red), cyclohexane (blue), or Isopar H (yellow).

2.2.2. Calculation of the electrostatic potential distribution of amine monomers

Geometry optimizations of all molecules and ions were performed by density functional theory (DFT) at the M06-2X level of theory with 6-311G(d) basis set,[1,2] including solvation energy corrections and Grimme's D3 (zero-damping) dispersion corrections.[3] The distribution of electrostatic potential and extreme points at the molecular van der Waals surface of all amine monomers (Figure S3) were calculated by Multiwfn 3.6 program.[4,5] All DFT calculations were conducted with the Gaussian 16 software package.[6]

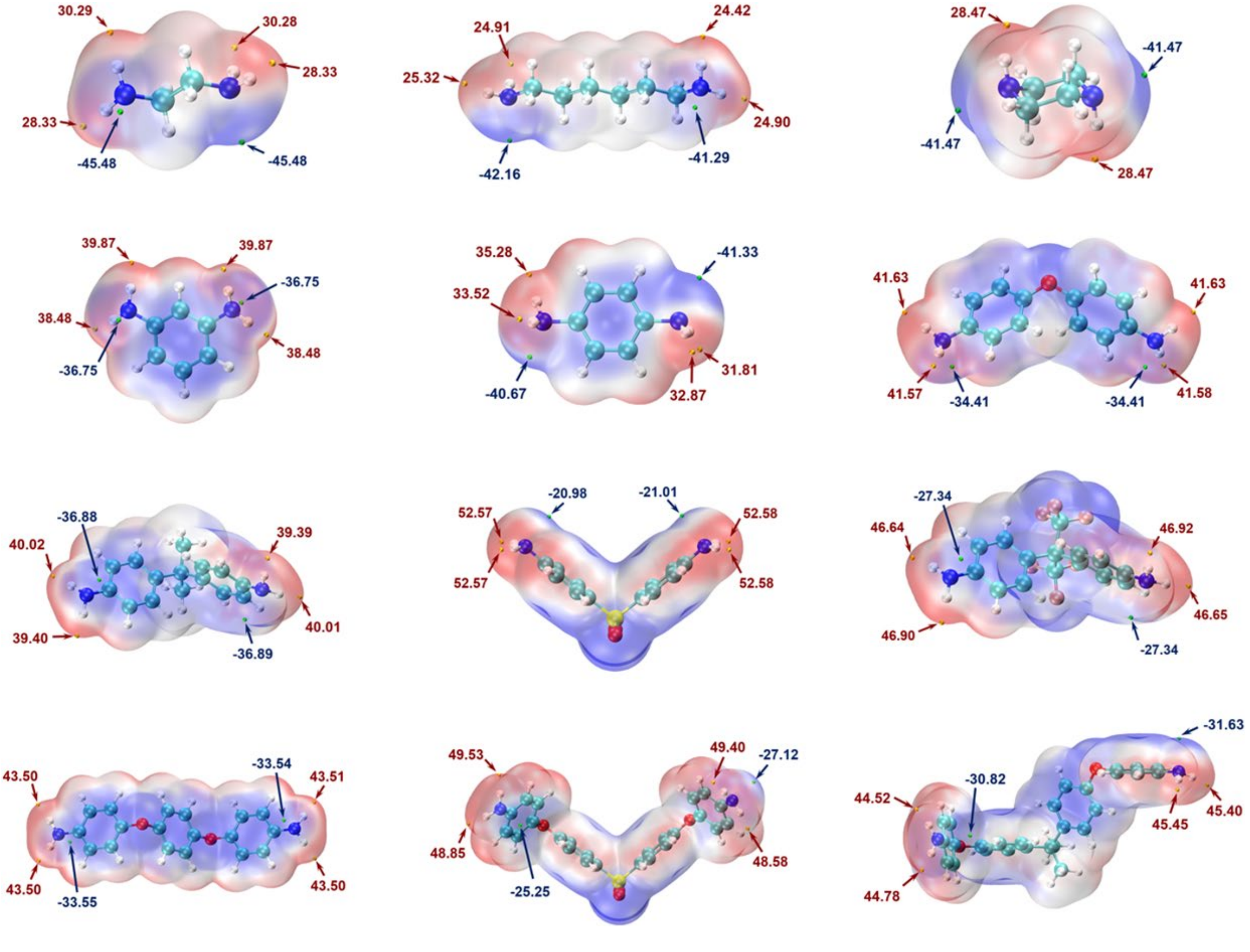

Figure S3. Distribution of electrostatic potential and corresponding extreme points at the molecular van der Waals surface of different amines studied in this work.

2.2.3. Simulation of the alkane-IL interface to determine the interfacial thickness and tension

The simulation box consists of a cube box with a length of 30 Å containing water molecules or several ion pairs of ILs in 30×30×15 Å and n-hexane molecules in 30×30×15 Å (Figure S4a). Fully flexible molecular all-atom models were based on DFT calculation. Parameters of the atomistic models based on the OPLS-AA force field were taken from Doherty. B et al., which can accurately describe the properties of ILs.[7-9] For water molecules, the three-site water model, spce was used.[10] Three-dimensional periodic boundary conditions (PBC) were used to avoid the influence of the box boundary during simulation. The cut-off distance of non-bonded interactions is 13 Å, and the long-range electrostatic interactions were calculated by the particle-mesh Ewald (PME) method.[11] Initial structures for molecular dynamic simulation were constructed by the Packmol software package.[12] Before the dynamics simulation, the steepest descent algorithm[13] was used to pre-equalize the system for eliminating the excessive stress in initial structures. After pre-equilibrium, a 10-ns NPT simulation with a time step of 2 fs was used to converge the system to the actual density. Then, a 40-ns production simulation under NPT ensemble with a time-step of 1 fs was carried out for data collection. Temperature and pressure coupling was performed using v-rescale thermostat[14] and Berendsen barostat[15] at 298 K and 1 atm. All simulations were carried out by using the GROMACS 2019.5 software package.[16] The interfacial scope for each alkane-IL interface was determined from Z-dependent density according to the “90% criterion” which is a common method to define interfacial layers.[17] Specifically, the interface position was defined as the coordinate corresponding to 10%-90% n-hexane density calculated from the density profiles (Figure S4b-h).

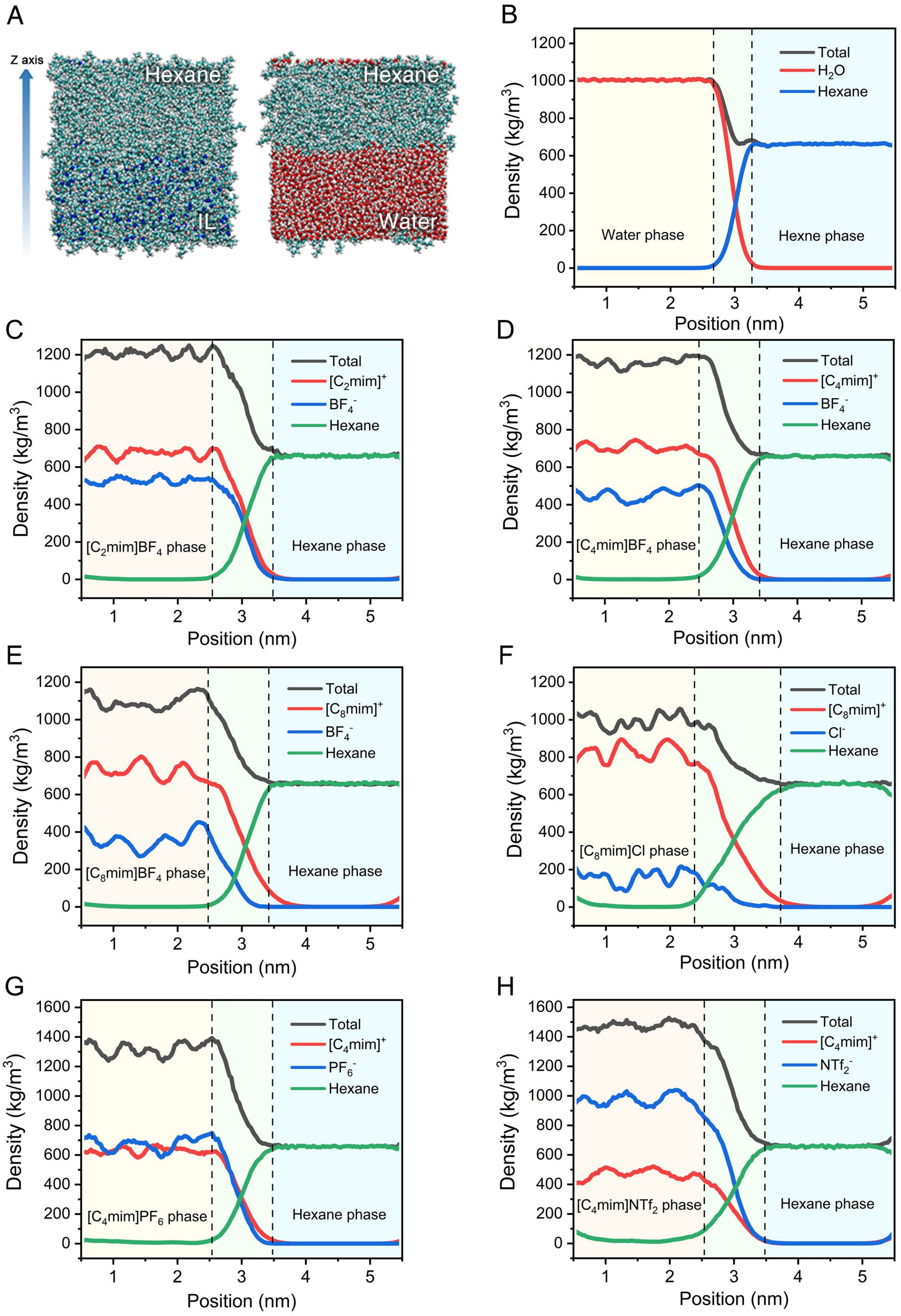

Figure S4. Species composition of the biphasic system and density distribution in the z-direction. a) Snapshots of the simulation boxes and schematic diagram of z-direction. b) hexane-water, c) hexane-$[C_2mim]BF_4$, d) hexane-$[C_4mim]BF_4$, e) hexane-$[C_8mim]BF_4$, f) hexane-$[C_8mim]Cl$, g) hexane-$[C_4mim]PF_6$, h) hexane-$[C_4mim]NTf_2$, respectively.

The interfacial tension was calculated by Equation S1:[18]

$$\gamma = \frac{1}{n} L_z \left[P_{zz} - \frac{1}{2}\left(P_{xx} + P_{yy}\right) \right] \qquad \text{(S1)}$$

where γ is the interfacial tension, n is the number of interfaces, L_z is the length of the simulated box along z-direction (Figure S4a). P_{xx}, P_{yy}, and P_{zz} are the partial pressures of the simulated box along the x, y, and z directions, respectively.

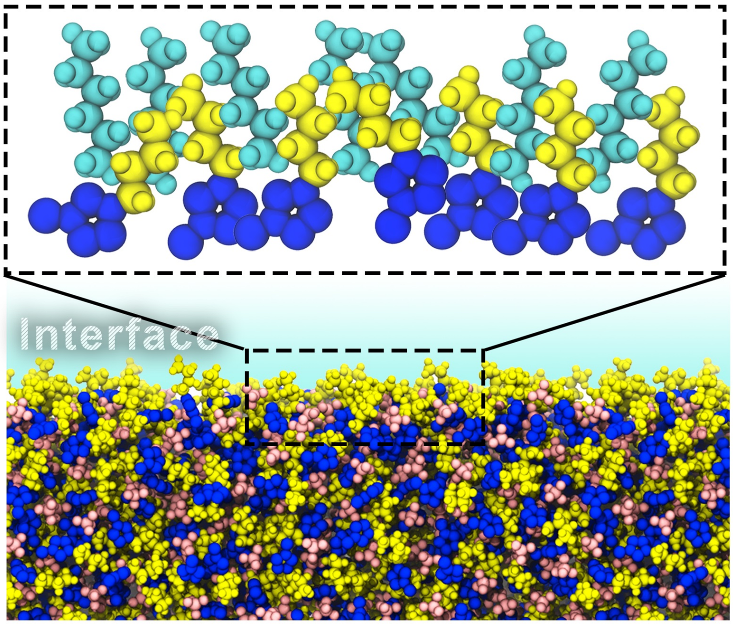

Figure S5. Orientation of 1-butyl-3-methylimidazole cation at the interface obtained by MD simulation. The cyan, blue, yellow, and pink spheres represent hexane, methylimidazole, butyl substituent, and tetrafluoroborate anion, respectively.

Interfacial tension was measured using the pendent drop technique by a Drop-Meter A-200 contact angle system (MAIST Vision Inspection & Measurement, China) at room temperature. IL was drawn into a syringe (2 mL in volume), and the syringe needle (1.8 mm in diameter) was inserted into n-hexane to squeeze a droplet that was about to leave the needle. The high-speed camera captured the shape of the drop, which can be used to calculate the interfacial tension. The average value was calculated from at least three parallel measurements.

Table S1. Interfacial tension between different ILs and n-hexane (mN m^{-1})

	n-Hexane	$[C_4mim]BF_4$	$[C_8mim]BF_4$	$[C_4mim]Cl$	$[C_8mim]Cl$	Water
Air (measured)	18.95	44.54	31.99	34.61	31.71	71.36
Hexane (measured)	-	14.34	3.47	5.64	3.35	51.22
Hexane (simulated)	-	20.61	5.42	6.74	4.33	51.74

2.2.4. Spatial distribution function of $[C_4mim]^+$ BF_4^- around amine monomers

The spatial distribution function based on MD simulations was used to infer the type and site of interaction between the IL and the amine. The simulation box consists of a cube box with a length of 50 Å containing an amine monomer and 420 pairs of $[C_4mim]^+$ and $BF4^-$. The amine monomer was confined in the center of the box for position restraint simulations. The remaining parameters and steps are the same as in 2.2.3. A 50-ns production simulation in the NPT ensemble at 298.15 K was carried out for data collection to obtain a smooth spatial distribution function (SDF) image. SDF was obtained by the TRAVIS program.[19] All visualization structures were provided by VMD software.[20]

2.2.5. Visualization of interfacial turbulence

The Marangoni effect is the phenomenon of macroscopic flow of liquids due to the presence of interfacial tension gradients. The contact of two immiscible solvents will produce an interfacial tension gradient in the interfacial polymerization process. The greater the interfacial tension gradient, the more intense the liquid turbulence. We used the following experiments to compare the degree of interface turbulence when IL and water contact with hexane. IL or water (10 mL) was added to a Petri dish with a diameter of 60 mm. Then the hexagonal polystyrene plastic sheets were floated on the surface of the liquid. A drop of hexane was added to the center of the liquid surface, and then the plastic sheets were pushed away because the hexane spreads on the surface of IL or water. The fluctuation of the interface was measured by directly photographing the disturbance of the interface (see Supplemental Movie) and observing the distance the plastic sheets were pushed away at the same time.

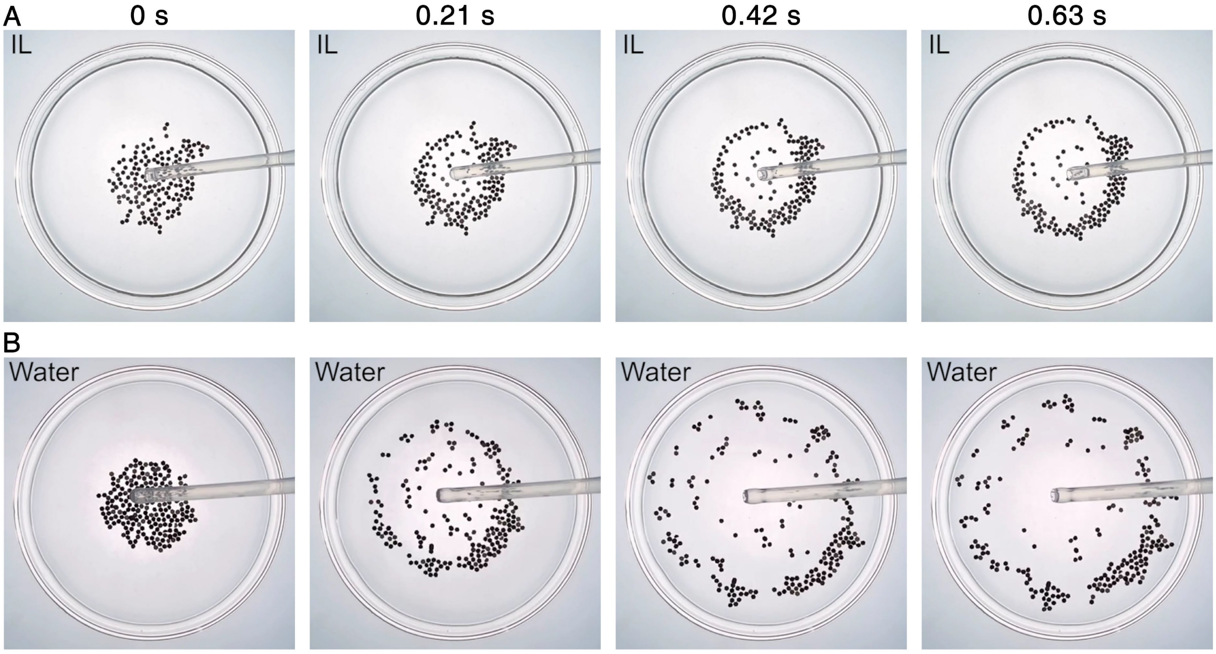

Figure S6. Visualization of interface turbulence. a-b) are the diffusion patterns of the plastic sheets on the surface when IL and water were in contact with hexane, respectively.

2.2.6. Discussion on the chemical stability of ILs

ILs are not fully inert.[21] In our work, amines are dissolved in [C_4mim][BF_4]. The most likely reaction between amine and IL at room temperature is the deprotonation of the second carbon (C2) on the imidazolium, and the resulting imidazole ring-opening. In fact, this reaction usually occurs between aliphatic tertiary amines[22] or inorganic bases (NaH)[23] and imidazolium-type ILs. The reaction caused by aromatic amines has not been reported yet. Even the secondary aliphatic amines have not been observed to react with such ILs.[24] The stable existence of amino-functionalized ILs also confirms the tolerance of imidazolium to amino to some extent.[25] It shows that only a strong base can pull out the proton on C2, but when the amino is not connected to a strong enough electron-donating group, its basicity is not enough to deprotonate C2. Besides, the basicity of aromatic amines is weaker than aliphatic amines due to p-π conjugation. Taking MPD as an example, DFT calculations were used to study the energy variation during the process of MPD attacking imidazolium C2 protons through flexible scanning (0.02 Å per step, 60 steps). All calculations are based on B3LYP-D3/6-311+G** level of theory in the implicit solvent model of [C_4mim][BF_4] (Table S2). The results show that the process also has no transition state and has a large energy barrier, which cannot occur at room temperature.

Table S2. Values of descriptors in SMD model of $[C_4mim][BF_4]$.

Descriptors	value
eps	61.69
epsinf	1.8374
HBondAcidity	0.06575
HBondBasicity	0.078
SurfaceTensionAtInterface	47.85
CarbonAromaticity	0.05
ElectronegativeHalogenicity	0.066675

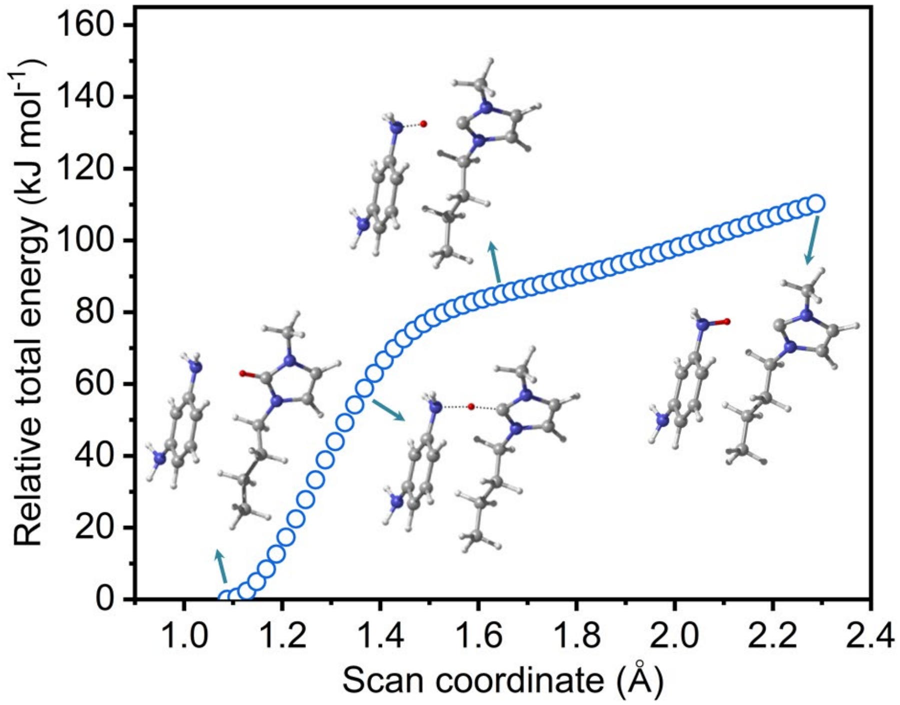

Figure S7. Total energy variation of the relaxed scan process for the deprotonation of $[C_4mim]^+$. The gray, white, blue, pink, and purple spheres represent carbon, hydrogen, nitrogen, boron, and fluorine, respectively. The proton involved in the reaction is marked in red.

2.2.7. Calculation of monomer partition coefficients in different solvent systems

The partition ratio K of a solute in two solvents can be obtained from the difference in free energy of the solute in the two solvents (Equation S2):

$$\Delta G = -RT \ln K \qquad \text{(S2)}$$

K is usually expressed in the form of the distribution coefficient logP (Equation S3):

$$\log P_{A/B} = \frac{\Delta G_{solv}(A) - \Delta G_{solv}(B)}{2.303RT} = \frac{E(A) - E(B)}{2.303RT} \qquad (S3)$$

where, $\log P_{A/B}$ is the partition coefficient of the solute between solvent A and solvent B, and ΔG_{solv} and E are the free energy and single point energy of the solute in the corresponding solvent, respectively. The single point energies were calculated by the self-consistent reaction field (SCRF) using the SMD model[26] at the M06-2X level of theory with the 6-31G(d) basis set.[27]

Table S3. The partition coefficient of monomers between different solvents.

Monomer	Solvent	E (Hartree)	logP (J mol^{-1})
MPD	Hexane	-342.811	-
	Water	-342.817	-2.8
	$[C_4mim]BF_4$	-342.818	-3.6
TMC	Hexane	-1950.743	-
	Water	-1950.73	4.4
	$[C_4mim]BF_4$	-1950.74	0.9

2.2.8. In situ monitoring of interfacial diffusion by UV spectroscopy

UV-Vis spectrophotometer (UV-2450, Shimadzu, Japan) was used to in situ monitor the interfacial diffusion process of amine and acyl chloride monomers in the transmission mode. UV-2450 has a UV incident beam with 1 mm wide and 12 mm high. Blackened stainless steel optical slits with a width of 30 μm and a length of 10 mm were mounted on the cuvette holders of the sample cell and reference cell to restrict the height of the UV beam entering the cuvettes. MPD and TMC were separately dissolved in $[C_4mim]BF_4$, water, and n-hexane, and the absorbance at 200-320 nm was measured to determine the characteristic absorption wavelength according to the Lambert-Beer law of each solute in different solvents. The phase interface between two liquids was constructed by adding 1.4 mL of $[C_4mim]BF_4$ (or water) and n-hexane to the quartz cuvette, respectively. Only one phase in the sample cell contains reactive monomers, while the reference cell contains no reactive monomers. The position of the sample cuvette was fine-tuned so that the UV beam passed right above or below the interface (see Figure S8). The kinetic mode in UVprobe software was used to in-situ monitor the absorbance

change at characteristic absorption wavelength caused by the interfacial diffusion of the monomers, with a sampling interval of 0.2 s and a test duration of 1 h. It is worth noting that we could not obtain the standard curve of absorbance-concentration of TMC in water because the dissolution of TMC in water is trace and accompanied by hydrolysis. We measured the water phase after TMC diffusion and found that the UV characteristic absorption wavelength is exactly the same as that of trimesic acid (TMA) in water. Therefore, we believe that it is not TMC but its hydrolyzed species that dissolve in water. We then used TMA to calibrate the concentration of TMC diffused into the water.

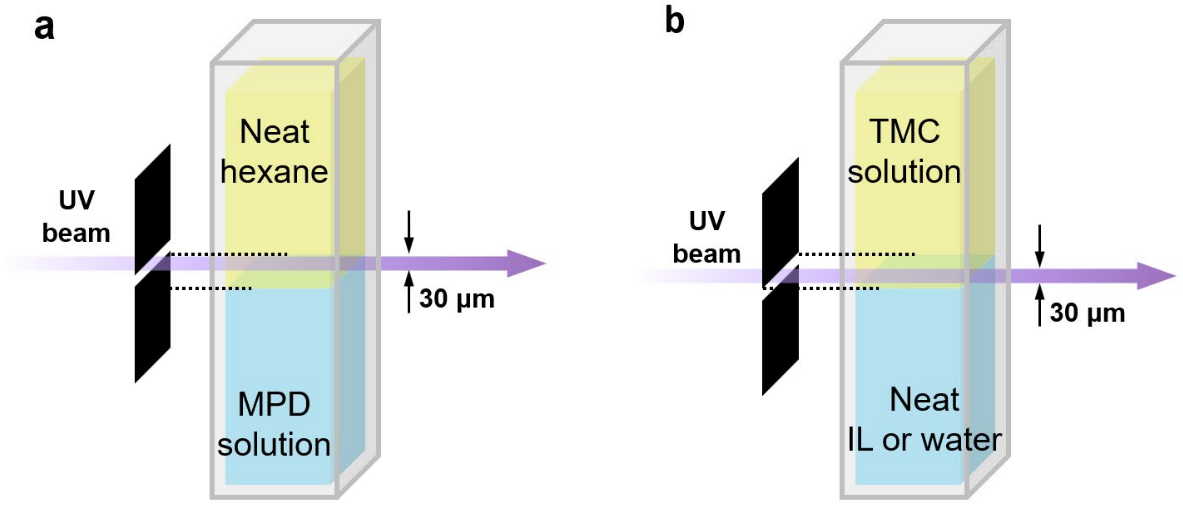

Figure S8. Schematic presentation of in situ monitoring of interfacial diffusion by UV spectroscopy. a) Monitoring MPD diffusion in the hexane phase. b) Monitoring the diffusion of TMC in the IL or water phase.

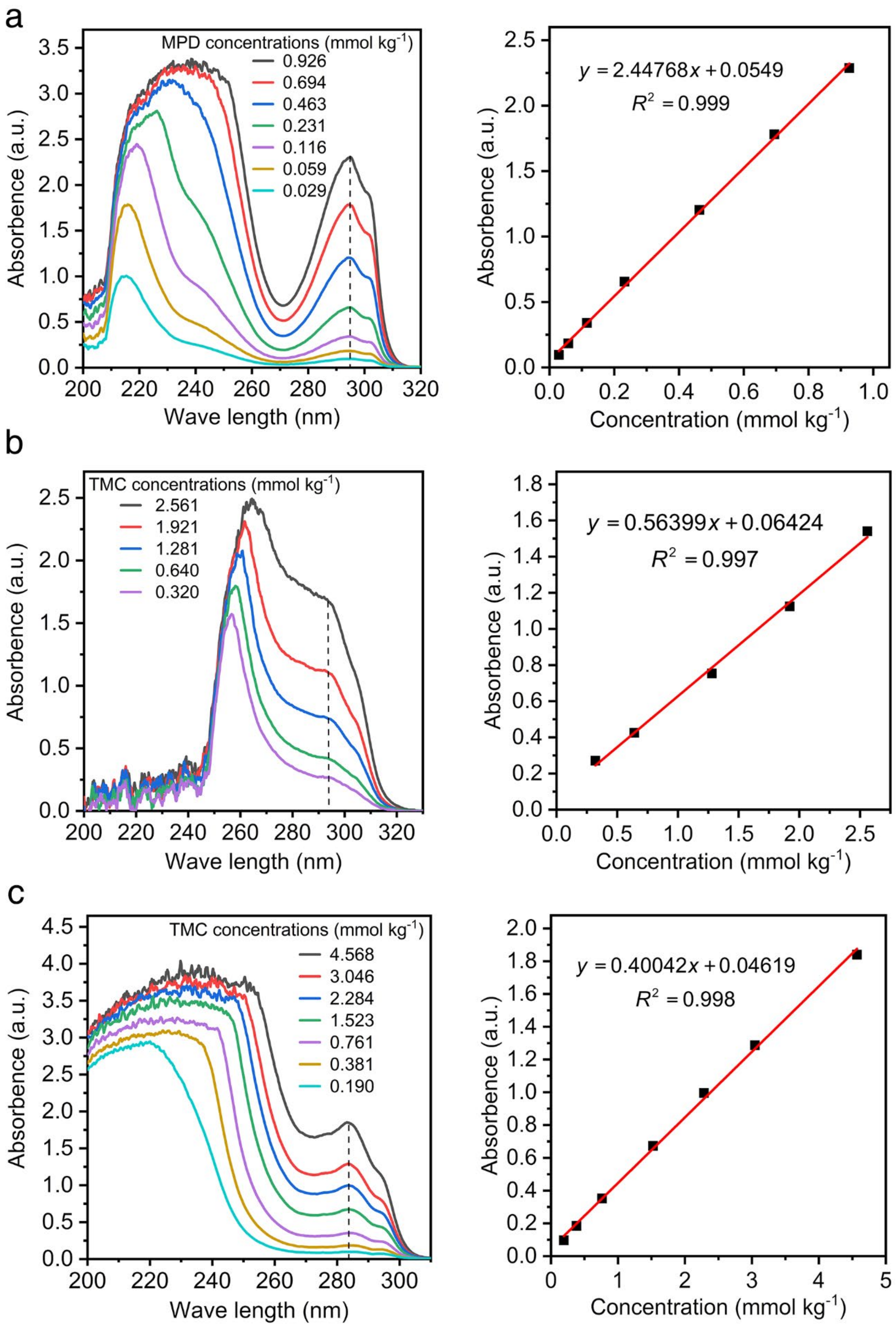

Figure S9. a-c) UV absorption peak and absorbance versus concentration standard curve of MPD in n-hexane, TMC in IL, and TMC in water, respectively.

2.2.9 Surface morphology, chargeability, and thickness of the resulting nanofilms

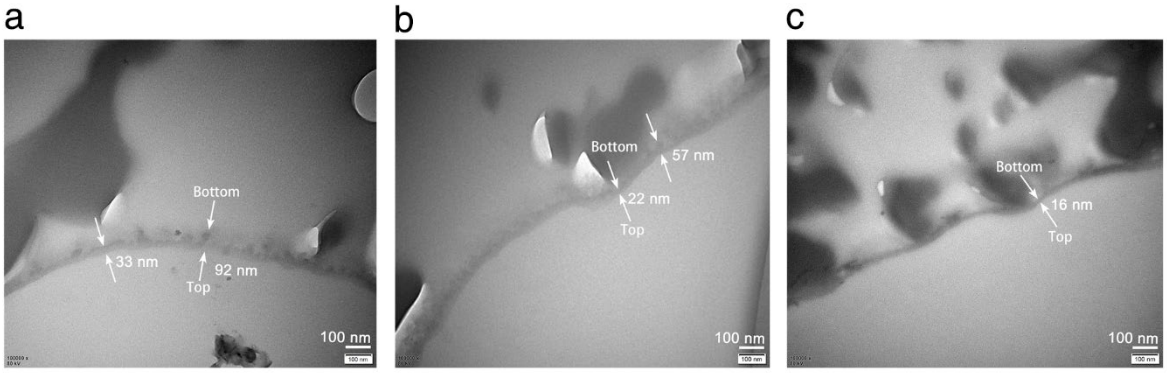

Figure S10. Raised structures and thicknesses of the polyamide nanofilms synthesized at different amine concentrations. a) 200 mM MPD, 5.6 mM TMC, 5 min. b) 300 mM MPD, 5.6 mM TMC, 5 min. c) 400 mM MPD, 5.6 mM TMC, 5 min.

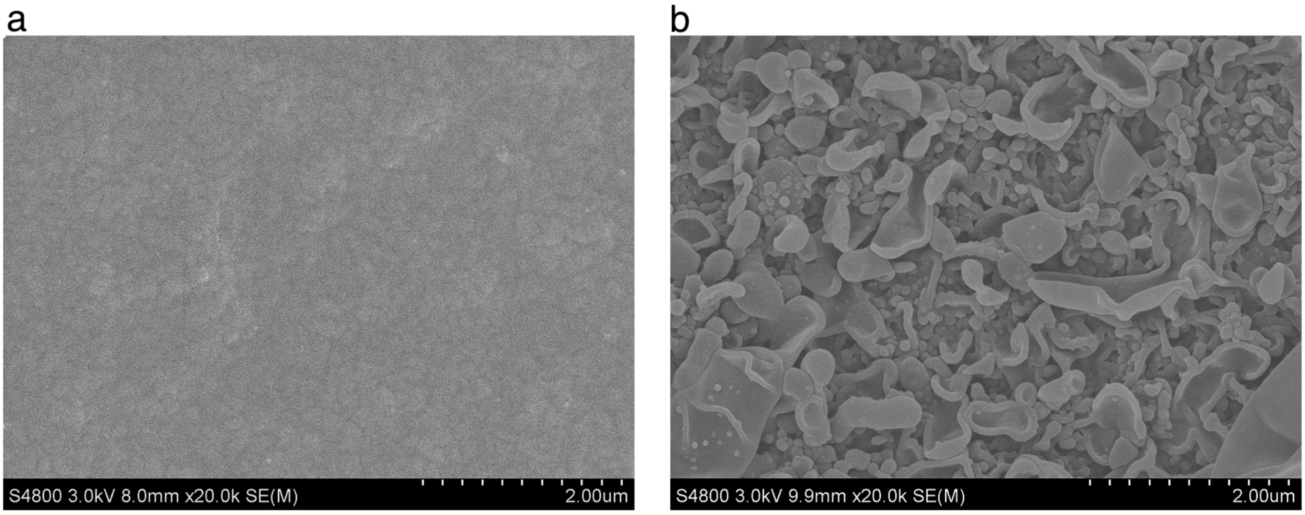

Figure S11. a-b) Surface morphologies of the polyamide nanofilms synthesized at the hexane-[C_4mim]BF_4 interface and the hexane-water interface (500 mM MPD, 5.6 mM TMC, 1 h).

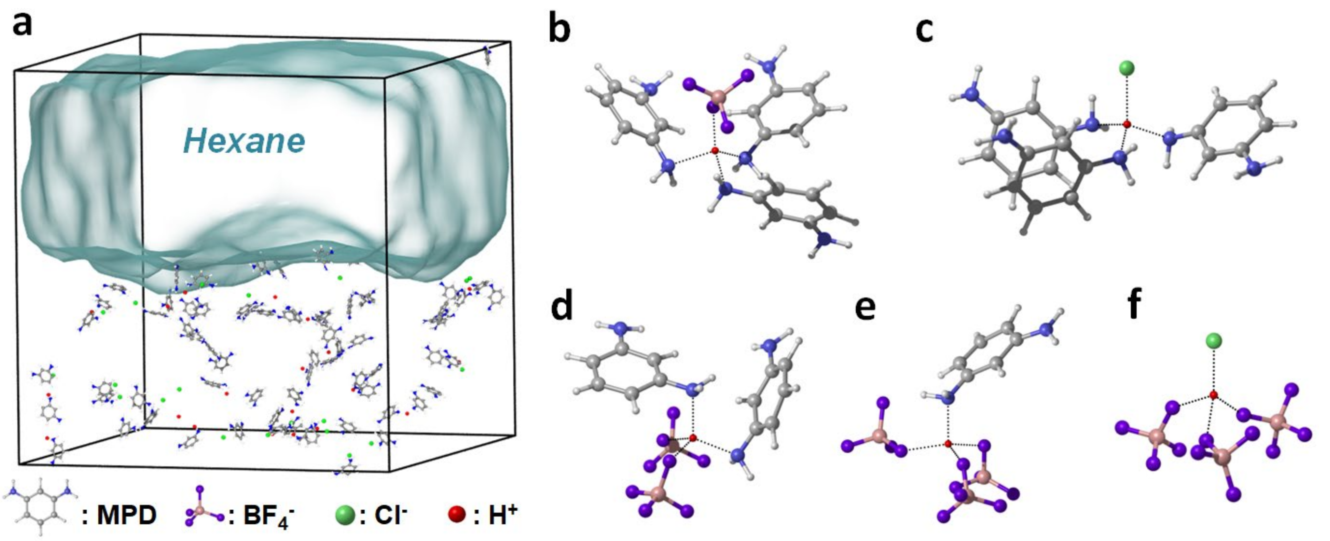

Figure S12. Distribution of byproduct H+ in hexane-[C_4mim]BF_4 interfacial system. a) A snapshot of simulation box that reflects the H+ distribution in hexane-[C_4mim]BF_4 interfacial system. The location distribution of hexane, MPD, and resulting H+ is only shown. b-f) Typical configurations extracted from the MD simulation. Note that it is not appropriate to use isolated protons in MD simulations, but we think it is acceptable to qualitatively study the approximate positions of protons in by this method.

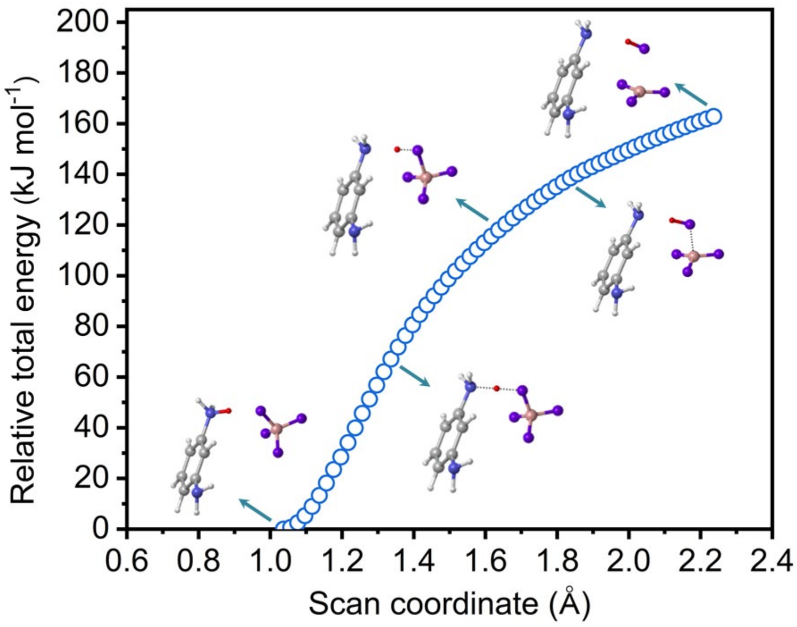

Figure S13. Total energy variation of the relaxed scan process for the acidolyzed of [BF_4]$^-$. The gray, white, blue, pink, and purple spheres represent carbon, hydrogen, nitrogen, boron, and fluorine, respectively. The proton involved in the reaction is marked in red. Calculation methods are the same as section 2.2.6. It can be seen that

this process has no transition state and has a large energy barrier. Regardless of thermodynamics or kinetics point of view, the resulting HCl after polymerization cannot form HF with $[BF_4]^-$.

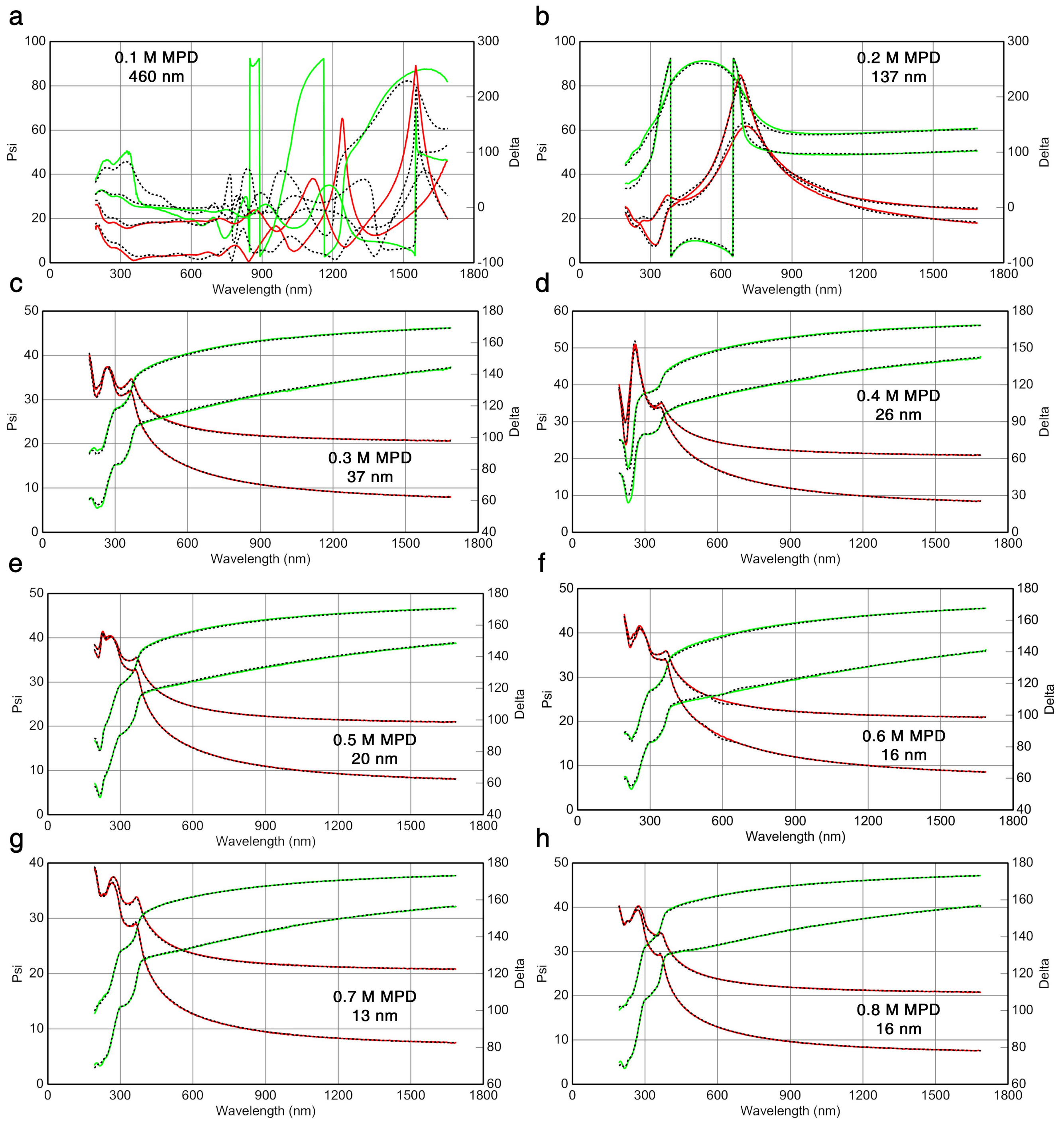

Figure S14. a-h) Elliptical polarization spectra of the polyamide nanofilms synthesized with different amine concentrations (reacted with 5.6 mM TMC, 10 min).

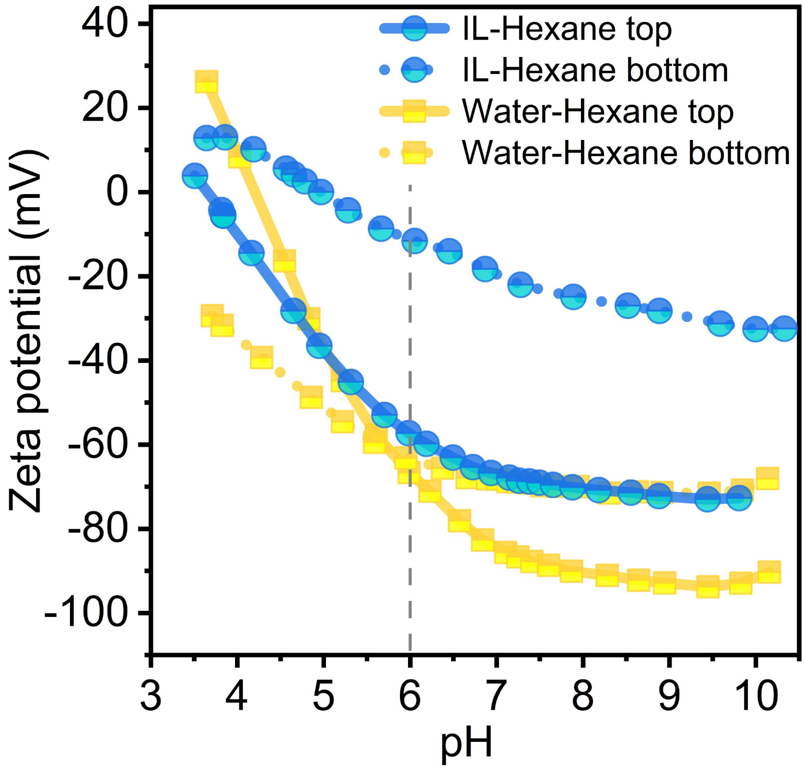

Figure S15. Zeta potentials on both sides of the polyamide nanofilms synthesized at the alkane-water interface and the alkane-IL interface (500 mM MPD, 0.56 mM TMC, 10 min).

2.2.10. Fabrication of thin-film composite membranes used for liquid phase separations

The amine monomer was sonicated in 2 mL of [C_4mim]BF_4, and 1 mL of the solution was taken into the interfacial polymerization device. One milliliter of TMC solution in n-hexane was added into the device along the edge with a pipette to carry out the interfacial polymerization. After reacting for 10 min, IL was suctioned off to make the formed polyamide nanofilm adhere to the AAO substrate. Then, n-hexane was poured off and the resulting composite membrane was heat-treated at 80 °C for 5 min. After that, the composite membrane was rinsed in ethanol to remove residual IL. The samples were stored in glycerin when the ethanol was not dry (see Figure S16). The concentrations of PIP and MPD in IL were 120 mᴍ and 500 mᴍ, respectively, and they were reacted with 0.56 mᴍ TMC in hexane for 10 min. For other aromatic amines, the concentration was 5 mᴍ and reacted with 0.0056 mᴍ TMC for 15 min.

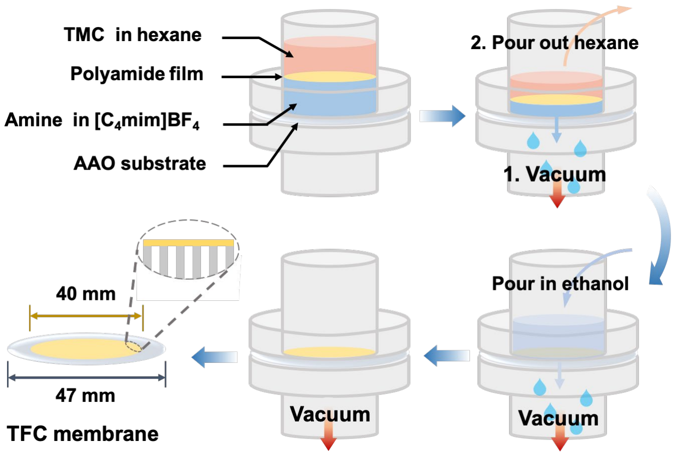

Figure S16. Schematic diagram for the preparation of thin-film composite membranes.

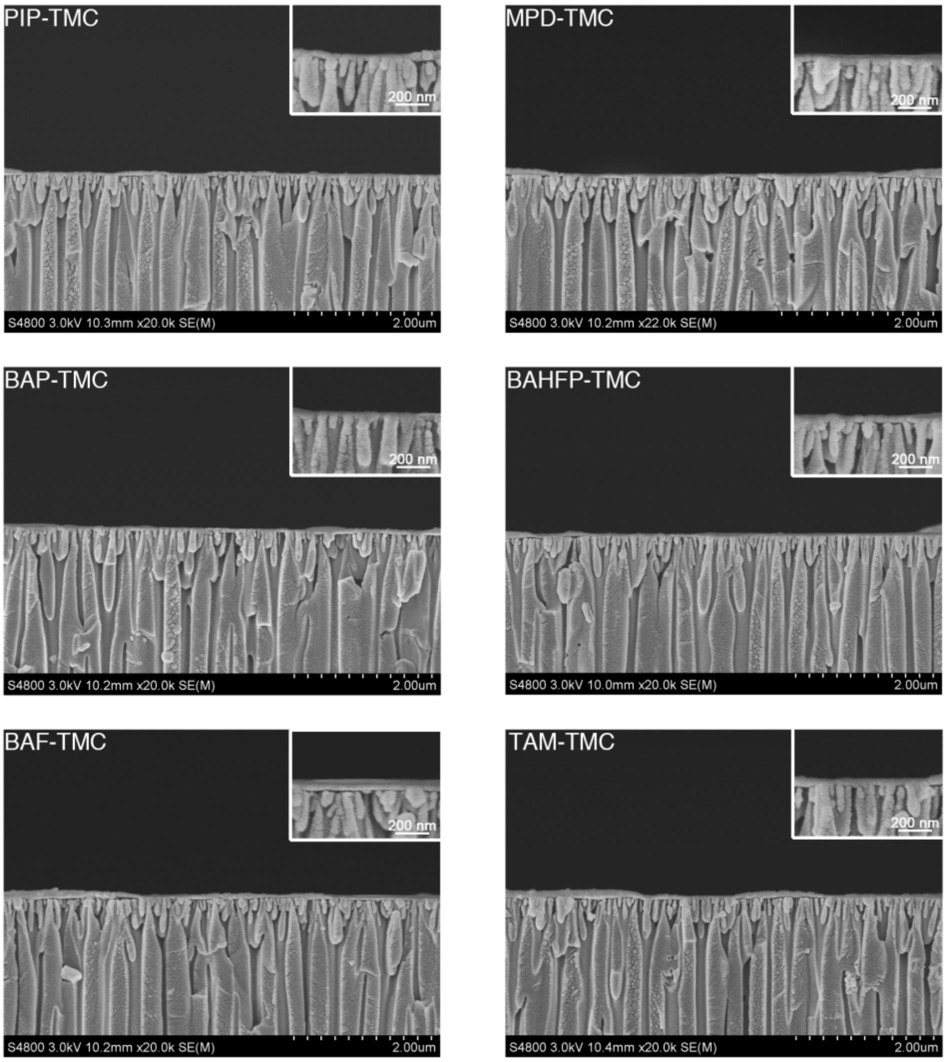

Figure S17. Cross-sectional FESEM images of ultra-thin interfacially polymerized polyamide nanofilms supported on AAO substrates. The nanofilms were synthesized by interfacial polymerization at the corresponding reaction condition described above.

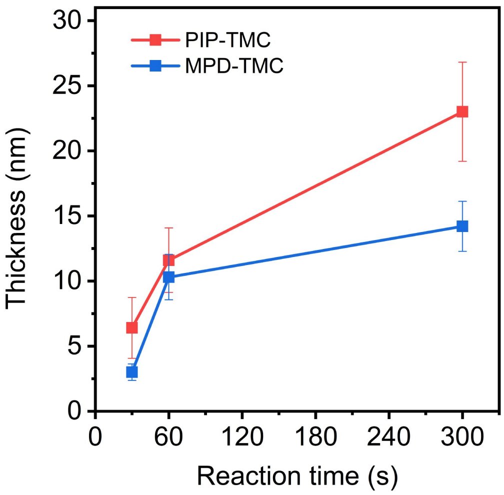

Figure S18. Thickness of the polyamide nanofilm as a function of reaction time (120 mM PIP, 0.56 mM TMC and 500 mM MPD, 0.56 mM TMC)

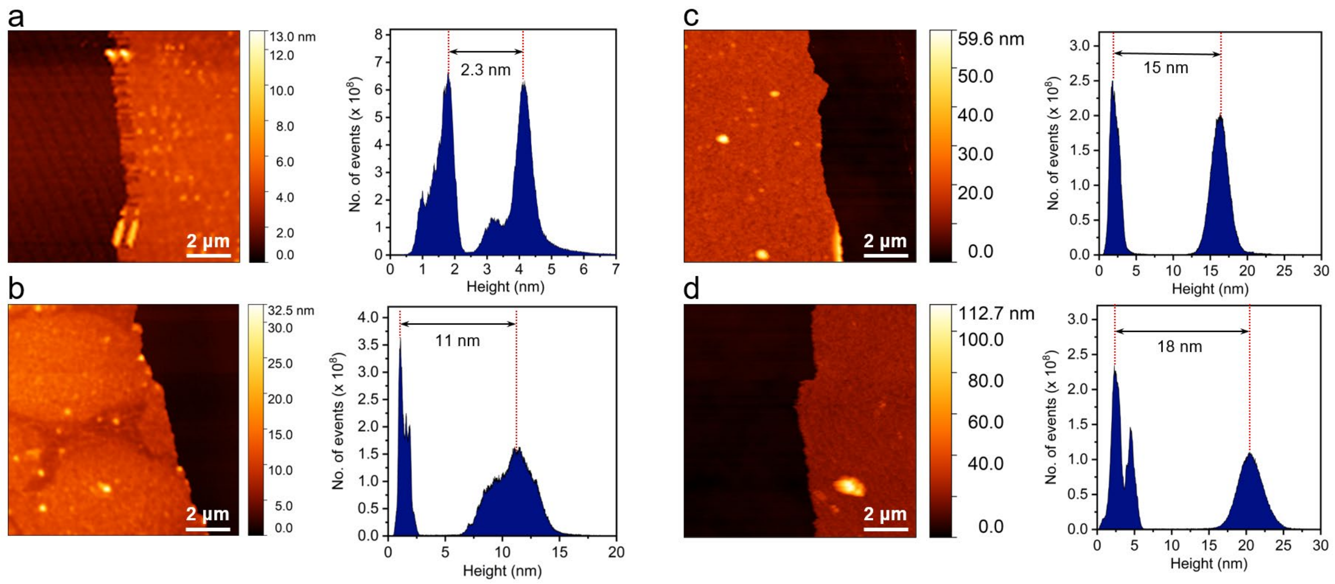

Figure S19. AFM height images and corresponding height profiles of polyamide nanofilms synthesized at hexane-[C_4mim]BF_4 interface with the reaction time of a) 30 s, b) 60 s, c) 300 s, and d) 3600 s (500 mM MPD, 0.56 mM TMC).

2.2.11. Aqueous nanofiltration and reverse osmosis

Thin-film composite membranes from the PIP-TMC and MPD-TMC nanofilms were used for aqueous nanofiltration and reverse osmosis, respectively, by a laboratory-scale crossflow unit with an effective membrane diameter of 3.0 cm. For the nanofiltration test, the samples were pre-compacted

by ultrapure water for 30 min under 0.8 MPa. Then, 2 L of 1000 ppm $MgSO_4$ aqueous solution was used as feed, and the operation pressure was kept at 0.6 MPa for measuring the water permeation flux and the conductivity of filtrate. In the reverse osmosis test, the pre-compacted and operation pressure were both 1.0 MPa, and the feed was 2000 ppm NaCl aqueous solution. During the test, the temperature of the feed was constant at 25 °C, and the cross-flow rate was fixed at 30 L h^{-1}. Water permeance (A) and salt rejection (R) were calculated by Equation S4 and Equation S5, respectively:

$$A = \frac{V}{S \times \Delta t \times p} \qquad (S4)$$

$$R = \left(1 - \frac{C_P}{C_f}\right) \times 100\% \qquad (S5)$$

where V is the volume of the permeate solution, p is the operation pressure used during the separation process, C_p and C_f are the concentrations of permeate and feed solutions detected by an electrical conductivity meter (Mettlertoledo, FE30, China), respectively. The solute permeability coefficient B was calculated by Equation S6:

$$B = Ap\left(\frac{1}{R} - 1\right) \qquad (S6)$$

Table S4. Data of water permeance and salt rejection for the polyamide-based composite membranes in Figure 4c.

Water permeance, A ($L\ m^{-2}\ h^{-1}\ bar^{-1}$)	Salt rejection (%)	Testing condition	Reference
13.1	98.5	2000 ppm $MgSO_4$ @4.8 bar	[28]
26.0	99.2	2000 ppm $MgSO_4$ @4.8 bar	
39.5	95.3	1000 ppm $MgSO_4$ @6 bar	[29]
14.7	75	1000 ppm $MgSO_4$ @6 bar	[30]
7.5	97	2000 ppm $MgSO_4$ @4 bar	[31]
12.0	98.3	1000 ppm $MgSO_4$ @5 bar	[32]
8.5	99.1	2000 ppm $MgSO_4$ @10 bar	[33]
29.8	86	1000 ppm $MgSO_4$ @6 bar	[34]
21.9	47.6	1000 ppm $MgSO_4$ @2.5 bar	[35]
28.0	96	2000 ppm $MgSO_4$ @10 bar	[36]
22.5	98	1500 ppm $MgSO_4$ @4 bar	[37]
16.4	97.8	1000 ppm $MgSO_4$ @3.5 bar	[38]
47.0	89.6	1000 ppm $MgSO_4$ @4 bar	[39]
33.0	94.1	1000 ppm $MgSO_4$ @6 bar	[40]

16.7	94.2	1000 ppm $MgSO_4$ @6 bar	[41]
17.6	94	1000 ppm $MgSO_4$ @6 bar	[42]
18.4	96.2	1000 ppm $MgSO_4$ @6 bar	[43]
30.6	93	2000 ppm $MgSO_4$ @6 bar	[44]
10.5	94	2000 ppm $MgSO_4$ @6 bar	[45]
19.1	19.4	500 ppm $MgSO_4$ @3 bar	[46]
17.0	80.9	1000 ppm $MgSO_4$ @6 bar	[47]
21.5	99.1	2000 ppm $MgSO_4$ @6 bar	[48]
53.5	80.7	1000 ppm $MgSO_4$ @2 bar	[49]
13.3	95	1000 ppm $MgSO_4$ @6 bar	[50]
2.4	96	2500 ppm $MgSO_4$ @5 bar	[51]
9.1	96.8	1000 ppm $MgSO_4$ @6 bar	[52]
0.7	94.2	2000 ppm $MgSO_4$ @10 bar	[53]
4.5	98.3	1000 ppm $MgSO_4$ @2 bar	[54]
20.0	98	1000 ppm $MgSO_4$ @3.45 bar	[55]
10.5	99.4	1000 ppm $MgSO_4$ @4 bar	[56]
19.3	71	1000 ppm $MgSO_4$ @2 bar	[57]
11.6	92	2000 ppm $MgSO_4$ @6 bar	[58]
11.2	70.5	1000 ppm $MgSO_4$ @10 bar	[59]
3.1	98	1000 ppm $MgSO_4$ @8 bar	[60]
17.3	80	500 ppm $MgSO_4$ @4 bar	[61]
3.8	70	1000 ppm $MgSO_4$ @6 bar	[62]
14.0	97	2000 ppm $MgSO_4$ @4 bar	[63]
10.9	97	2000 ppm $MgSO_4$ @4.8 bar	[64]
8.9	98.7	2000 ppm $MgSO_4$ @4.8 bar	
6.6	98	2000 ppm $MgSO_4$ @7.58 bar	[65]
9.9	98	2000 ppm $MgSO_4$ @5.17 bar	[66]
5.2	98	2000 ppm $MgSO_4$ @7.58 bar	[67]
4.8	98	2000 ppm $MgSO_4$ @7.58 bar	[68]
5.9	97	2000 ppm $MgSO_4$ @5.2 bar	[69]
6.0	99.8	2000 ppm $MgSO_4$ @7.58 bar	[70]
32.6	99.2	1000 ppm $MgSO_4$ @6 bar	This work
29.7	99.4	1000 ppm $MgSO_4$ @6 bar	This work
24.5	99.4	1000 ppm $MgSO_4$ @6 bar	This work

Table S5. Data of water permeance and salt rejection for the polyamide-based composite membranes in Figure 4d.

Water permeance, A (L m^{-2} h^{-1} bar^{-1})	Salt rejection (%)	Testing condition	Reference
14.7	94	2000 ppm NaCl @15.5 bar	[71]
3.7	95	2000 ppm NaCl @15.5 bar	
2.9	97.5	2000 ppm NaCl @15.5 bar	
0.8	95	2000 ppm NaCl @20 bar	[72]
2.7	96	2000 ppm NaCl @40 bar	
4.1	93.3	2000 ppm NaCl @20 bar	
1.4	95.7	2000 ppm NaCl @15.5 bar	[73]
1.3	98.7	2000 ppm NaCl @15.5 bar	
1.5	98.2	2000 ppm NaCl @15.5 bar	[74]
0.6	96	2000 ppm NaCl @15.5 bar	
2.1	99.1	2000 ppm NaCl @15.5 bar	[75]
0.5	99	2000 ppm NaCl @15.5 bar	
1.5	99.1	2000 ppm NaCl @15.5 bar	[76]
5.4	98.2	2000 ppm NaCl @24.1 bar	[77]
2.4	97.0	2000 ppm NaCl @15.5 bar	[78]
2.8	98.6	2000 ppm NaCl @16 bar	[79]
1.8	99.1	2000 ppm NaCl @15.5 bar	[80]
4.4	95	2000 ppm NaCl @20.7 bar	[81]
3.0	98.3	50 mM NaCl @27.6 bar	[82]
6.4	93	2000 ppm NaCl @10 bar	[53]
0.6	99.4	2000 ppm NaCl @15.5 bar	[83]
2.0	95.6	2000 ppm NaCl @15 bar	[84]
1.5	99	2000 ppm NaCl @20 bar	[85]
2.7	98	2000 ppm NaCl @16 bar	[86]
1.2	94	2000 ppm NaCl @21 bar	[87]
1.7	92	2000 ppm NaCl @15 bar	[88]
3.3	95.3	1000 ppm NaCl @2 bar	[89]
0.5	99.82	32000 ppm NaCl @55 bar	[90]
2.9	99.5	2000 ppm NaCl @15.5 bar	[91]
2.9	99.5	2000 ppm NaCl @15.5 bar	[92]
1.8	99.5	2000 ppm NaCl @13.79 bar	[93]
1.5	99	2000 ppm NaCl @29.3 bar	[94]
3.2	99.5	2000 ppm NaCl @15.5 bar	
6.2	99	500 ppm NaCl @7.93 bar	
5.4	95	500 ppm NaCl @8.62 bar	[95]
0.5	99.8	32000 ppm NaCl @55 bar	[96]

2.9	99.5	2000 ppm NaCl @15.5 bar	[97]
5.0	99.4	2000 ppm NaCl @10.5 bar	[98]
2.9	99.7	2000 ppm NaCl @15.5 bar	[99]
2.8	95.7	2000 ppm NaCl @15.5 bar	[100]
10.1	85.7	2000 ppm NaCl @10 bar	This work
4.8	99.2	2000 ppm NaCl @10 bar	This work
6.9	98.9	2000 ppm NaCl @10 bar	This work

2.2.12. Elemental composition and crosslinking degree of the polyamide nanofilms

Gold-coated silicon wafers were used as the substrate in XPS analysis to eliminate the interference of silicon dioxide to determine the cross-linking degree more reliable. The degree of network cross-linking (DNC) was calculated from the narrow scanning spectra of O1s and N1s. Suppose there are X cross-linked structures and Y linear structures, then there are 3X oxygen atoms and 2X nitrogen atoms in each cross-linked polyamide structure. Because the unreacted acyl chloride groups in the linear structure are hydrolyzed to carboxyl groups, there are 4Y oxygen atoms and 2Y nitrogen atoms in each linear structure, so the O/N ratio in the polyamide nanofilms can be expressed as Equation S7:

$$\mathrm{O/N} = \frac{\mathrm{3X+4Y}}{\mathrm{3X+2Y}} \times 100\% \qquad \text{(S7)}$$

The DNC can be calculated by Equation S8:

$$\mathrm{DNC} = \frac{\mathrm{X}}{\mathrm{X+Y}} \qquad \text{(S8)}$$

The XPS spectra and the analysis results are shown in Figure S20, S21, and Table S6.

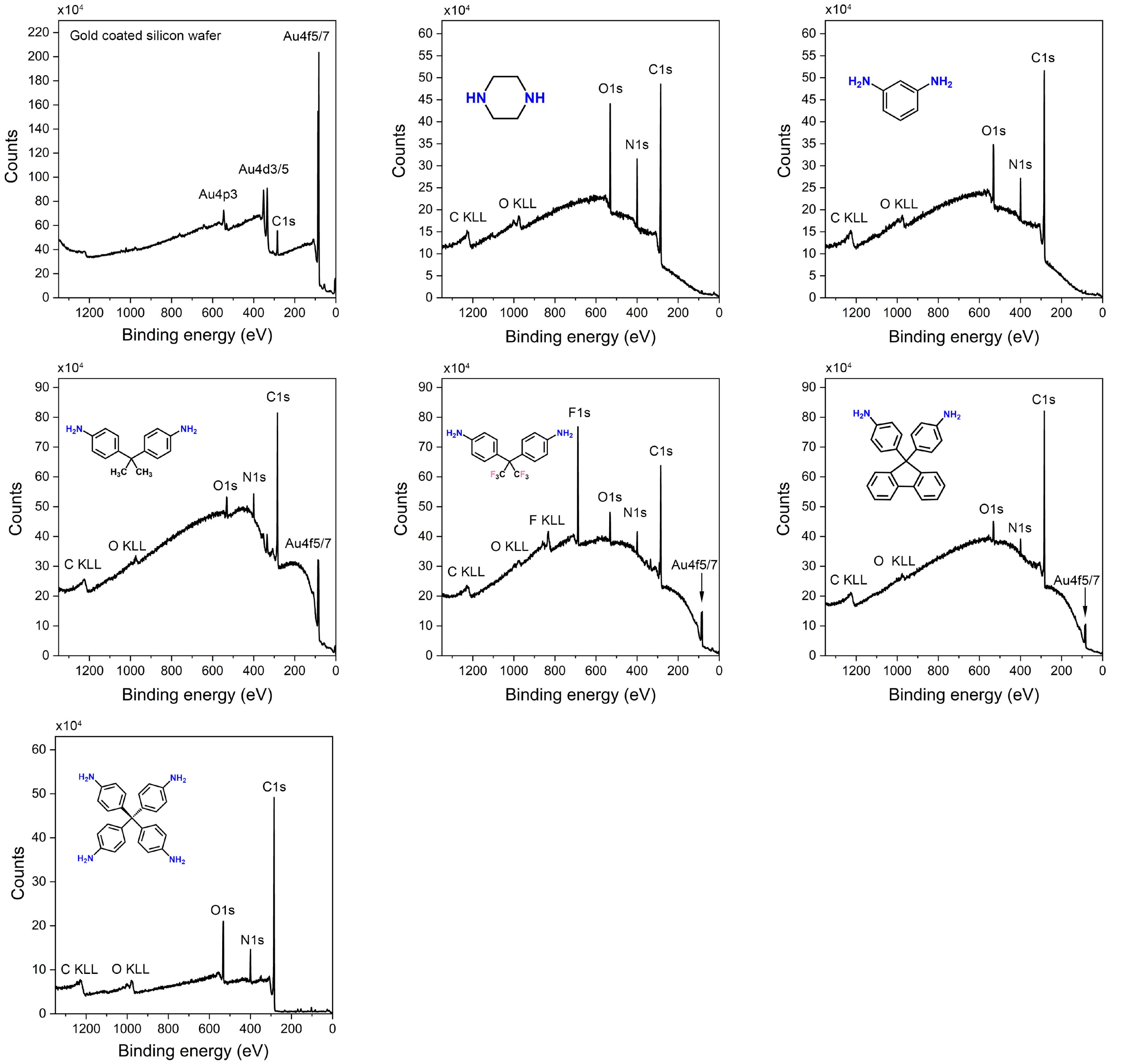

Figure S20. XPS survey spectra of the polyamide nanofilms transferred to gold-coated silicon wafers.

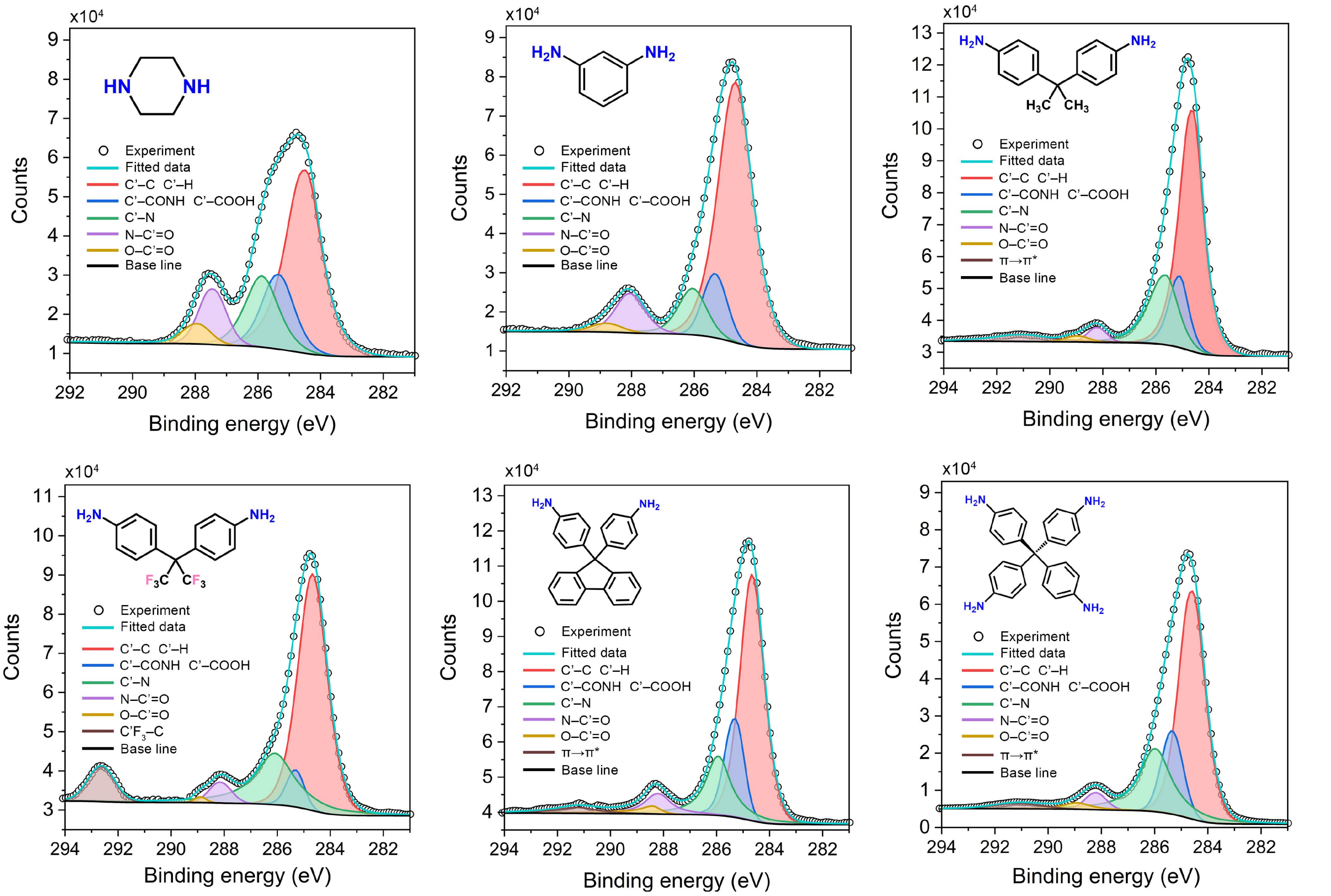

Figure S21. Narrow scan results of X-ray photoelectron C1s spectra of the polyamide nanofilms transferred to gold-coated silicon wafers.

Table S6. O/N ratio and DNC of the polyamide nanofilms.

Polyamide nanofilm	Peak	Peak position (eV)	FWHM (eV)	Aera (CPS.eV)	Atomtic (%)	O/N ratio	DNC (%)
MPD-TMC	N1s	399.67	1.43	31567.47	48.47	1.06	90.8
	O1s	531.01	1.48	52309.3	51.53		
PIP-TMC	N1s	399.75	1.38	47026.1	49.36	1.03	96.2
	O1s	531.01	1.55	75209.85	50.64		
BAP-TMC	N1s	399.68	1.37	25442.07	47.09	1.12	82.5
	O1s	531.04	1.72	44549.89	52.91		
BAHFP-TMC	N1s	399.97	1.49	22952.98	45.88	1.17	75.3
	O1s	531.35	1.93	42196.34	54.12		
BAF-TMC	N1s	399.65	1.4	19534.45	47.14	1.12	82.8
	O1s	531.09	2.06	34140.55	52.86		
TAM-TMC	N1s	399.45	1.73	23985.65	43.69	1.29	-
	O1s	531.22	2.09	48157.78	56.31		

2.2.13. Structure information of the crosslinked polyamide networks

We invoked computer simulations to reveal the structure information of the synthesized polyamide networks. The initial simulated box was a cubic box with a length of 70 Å, in which two repeating units were filled at a density of 0.4 g cm^{-3}. The Polymatic[101] program was then used to simulate the crosslinking process with the polymer consistent force field (pcff). The degree of crosslinking was set to 100% to study the intrinsic structures of such polyamides. After the crosslinking was completed, the uncrosslinked residues were manually completed, in which the acyl residue was added to a carboxyl group, and the amino residue was completed by a hydrogen atom. Then, a 21-step simulated annealing process was performed on the completed structure through the LAMMPS program to eliminate residual internal stress in the structure. Structural information such as the pore size distribution and the accessible specific surface area of the polyamide was calculated by the ZEO++ software package based on the Voronoi decomposition.[102,103] The molecular volume and the surface area of different monomers on the isosurface of 0.01 were calculated by the Multiwfn program using the Marching Tetrahedron algorithm.[4,104]

Table S7. Simulated structure information of porous polyamides synthesized by IP@AILI with different monomer compositions.

Polyamide nanofilm	Volume (Å^3)	Density (g cm^{-3})	Global cavity diameter (Å)	Pore limiting diameter (Å)	Largest cavity diameter (Å)	Surface area 0.86 (m^2 g^{-1})	Surface area 1.55 (m^2 g^{-1})
MPD-TMC	103730	1.3260	4.60	1.75	4.00	775.2	26.12
BAP-TMC	120239	1.1643	5.30	1.76	4.33	1158.3	66.4
BAHFP-TMC	99664	1.4244	5.56	1.75	4.31	937.2	67.2
BAF-TMC	121082	1.1739	4.84	1.84	4.84	1280.9	95.0
TAM-TMC	116894	1.2059	7.11	2.01	5.63	1385.9	178.8

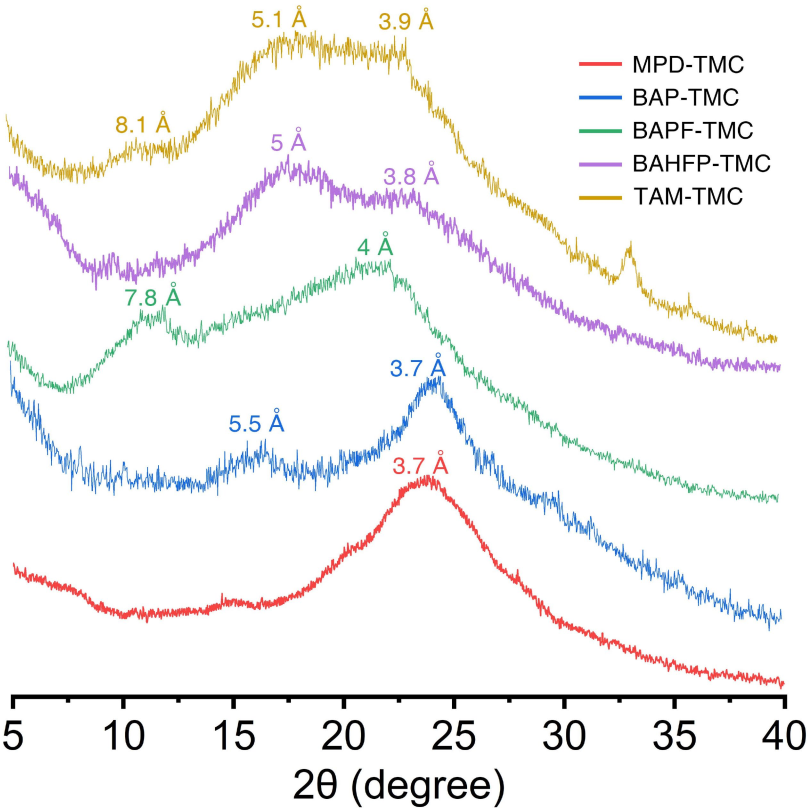

Figure S22. Wide-angle X-ray scattering pattern of different polyamide powders.

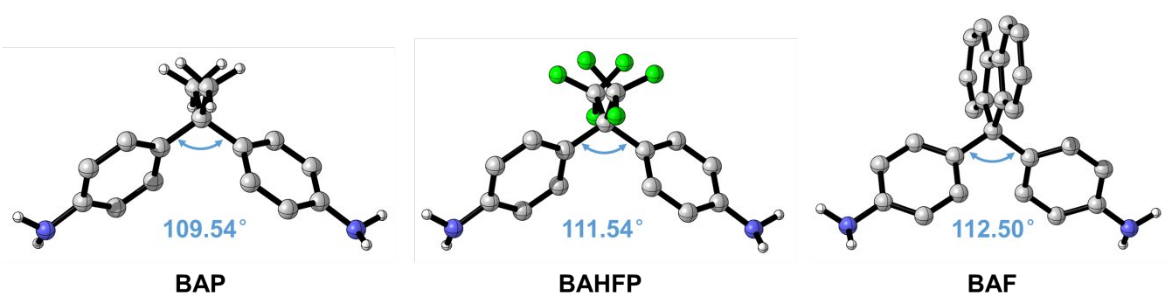

Figure S23. Influence of the side group size of BAP, BAHFP, and BAF on their spatial topology.

Table S8. Molecular volume and surface area of BAP, BAHFP, and BAF (0.01 iso-surface).

Monomer	Molecular volume (Å^3)	Molecular surface area (Å^2)
BAP	313.1	277.7
BAHFP	333.1	292.3
BAF	434.7	374.7

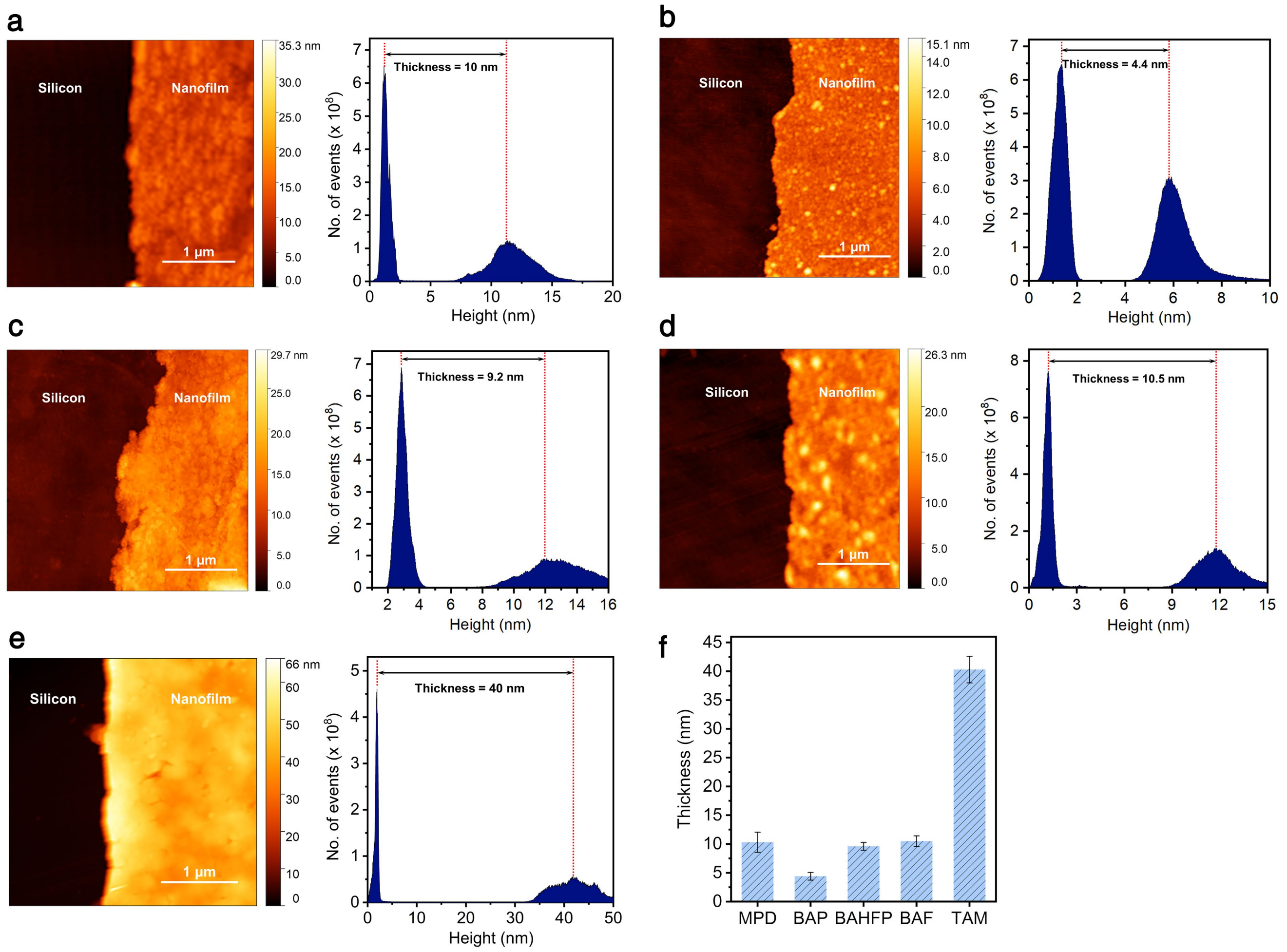

Figure S24. AFM height images and corresponding height profiles of a section of polyamide nanofilms synthesized for organic solvent nanofiltration and gas separation. a-e) MPD-TMC, BAP-TMC, BAHFP-TMC, BAF-TMC, and TAM-TMC, respectively. f) Thickness statistics of the polyamide nanofilms.

2.2.14. Organic solvent nanofiltration

Organic solvent nanofiltration experiments were carried out in a dead-end device with an effective filtration area of 12.56 cm^2 and a test pressure of 3 bar provided by a nitrogen cylinder. Typically, a 50 mL solution of dyes in methanol was used as the feed with a concentration of 25 ppm. Before testing, the device was maintained at a pressure of 0.5 bar for 30 min to ensure that the polyamide nanofilm reached the adsorption equilibrium for the dye. The solvent permeance and solute rejection were calculated using Equation 2 and 3, respectively. The solute concentration in a solvent was measured by a UV spectrophotometer.

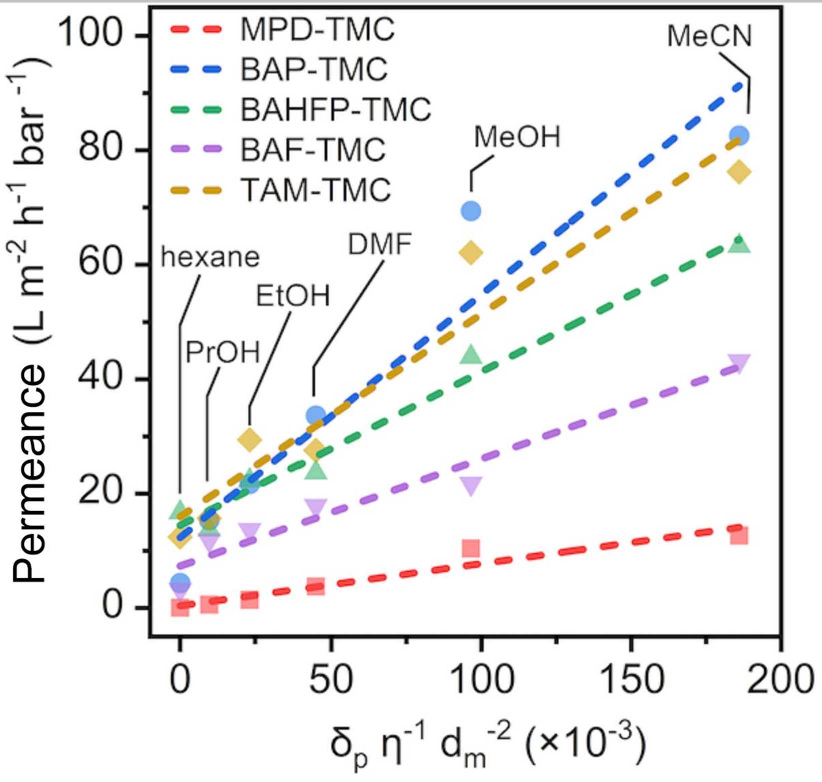

Figure S25. The plot of solvent permeance against the combined solvent property (viscosity, molar diameter, and solubility parameter) for the five polyamide composite membranes.

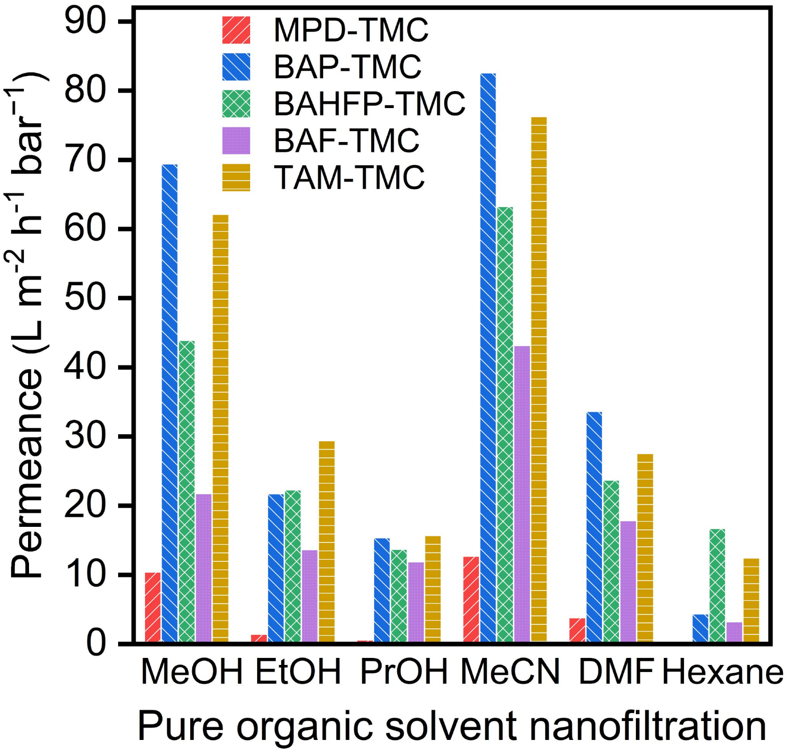

Figure S26. Pure organic solvent permeation flux of the polyamide composite membranes.

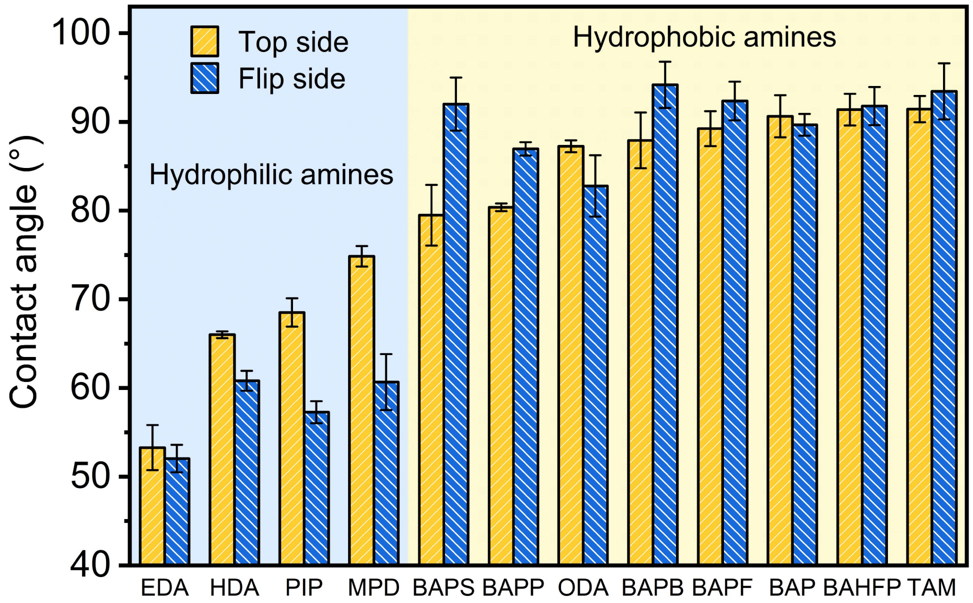

Figure S27. The water contact angle of the polyamide nanofilms synthesized by different amines.

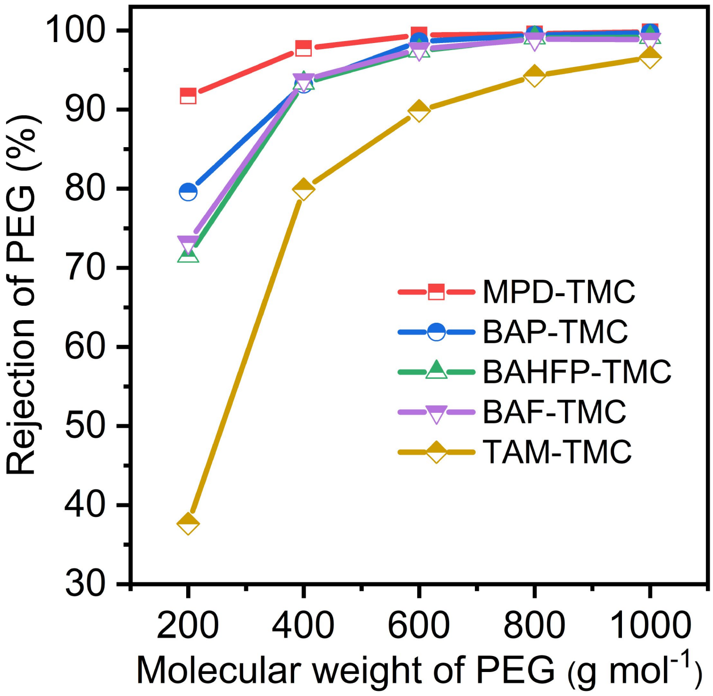

Figure S28. PEG retention of the polyamide composite membranes.

Table S9. Data of methanol permeance and molecular rejection (Mw=320-410 g mol^{-1}) for organic solvent nanofiltration membranes in Figure 5d.

Methanol permeance (L m^{-2} h^{-1} bar^{-1})	Rejection (%)	Solute	Reference
0.2	92	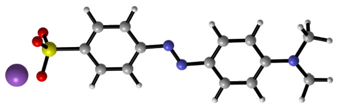 Methyl orange Mw=327.33	[105]
1.8	95		
5.8	93		
6.1	94		
9.6	91		
74.2	88.5	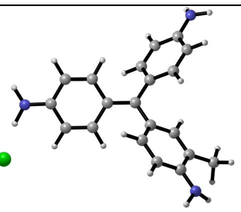 Basic fuchsine Mw=337.86	[106]
224.75	41.3	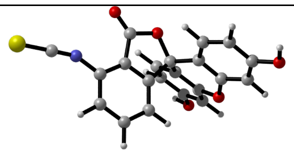 Fluorescein-4-isothiocyanate Mw=389.38	[107]
2.39	94.8	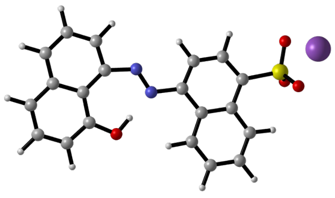 Naphthalene brown Mw=400.38	[108]
8.98	98.9		
12.97	98.9		
2.97	99.7		
3.74	96.4		
1.49	96.9		
4.66	97.5		

8	97		
6	98.7		[109]
0.6	97.7		
0.6	99.7		
66	75	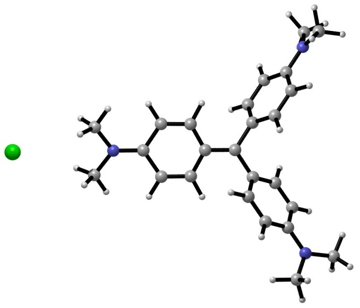	
47.6	83		[110]
17.6	88		
2.45	92		
10.42	98.3	Crystal violet	
69.42	96.9	Mw=407.99	
43.89	93.5		This work
21.76	96.7		
62.13	87.2		

2.2.15. Fabrication of freestanding polyamide nanofilms and gas separation membranes

First, polydimethylsiloxane (PDMS) (1 wt%), tetraethoxysilane (TEOS) (0.5 wt%), and dibutyltin dilaurate (DBTL) (0.5 wt%) were dissolved in Isopar H (50 mL), stirred for 3 min, and then left to cure for 1 h. The cross-linked PDMS was spin-coated on an AAO substrate (1500 rpm, 1 min). The amine monomer was sonicated in 2 mL of [C_4mim]BF_4, and 1 mL of the solution was taken into the interfacial polymerization device. 1 mL n-hexane solution of TMC was taken into the device along the edge with a pipette to carry out the interfacial polymerization. After reacting for 10 min, the liquid in the device was removed as much as possible by a pipette, and the nanofilm was attached to the wire mesh below. The nanofilm loaded on the polystyrene substrate was rinsed in ethanol to remove residual IL and amine monomer. The nanofilm was then free to float on the water surface and picked up with a crosslinked PDMS-coated AAO disc (see Figure S29 and S30).

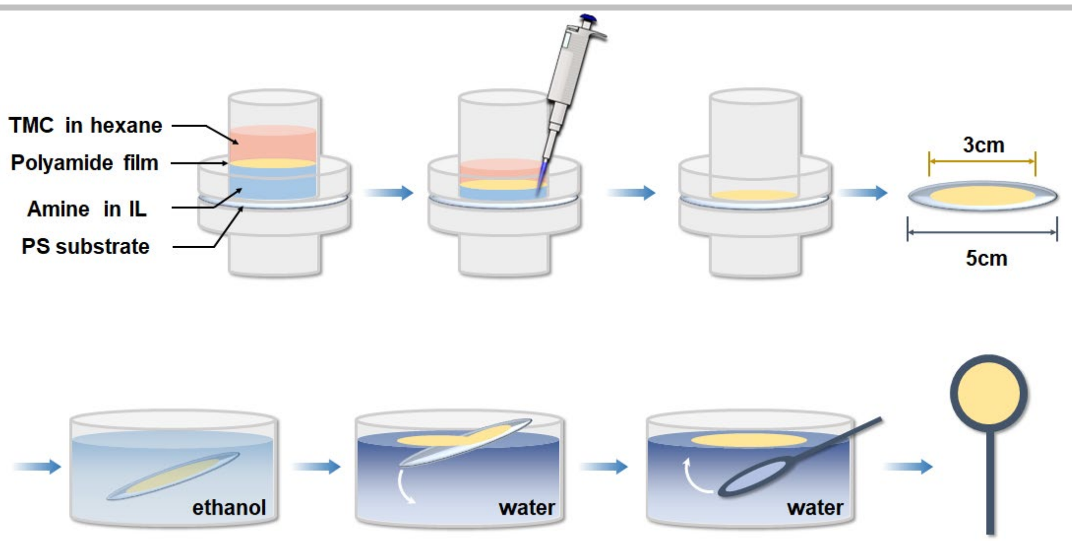

Figure S29. Schematic diagram of the preparation of freestanding polyamide nanofilm and gas separation membrane.

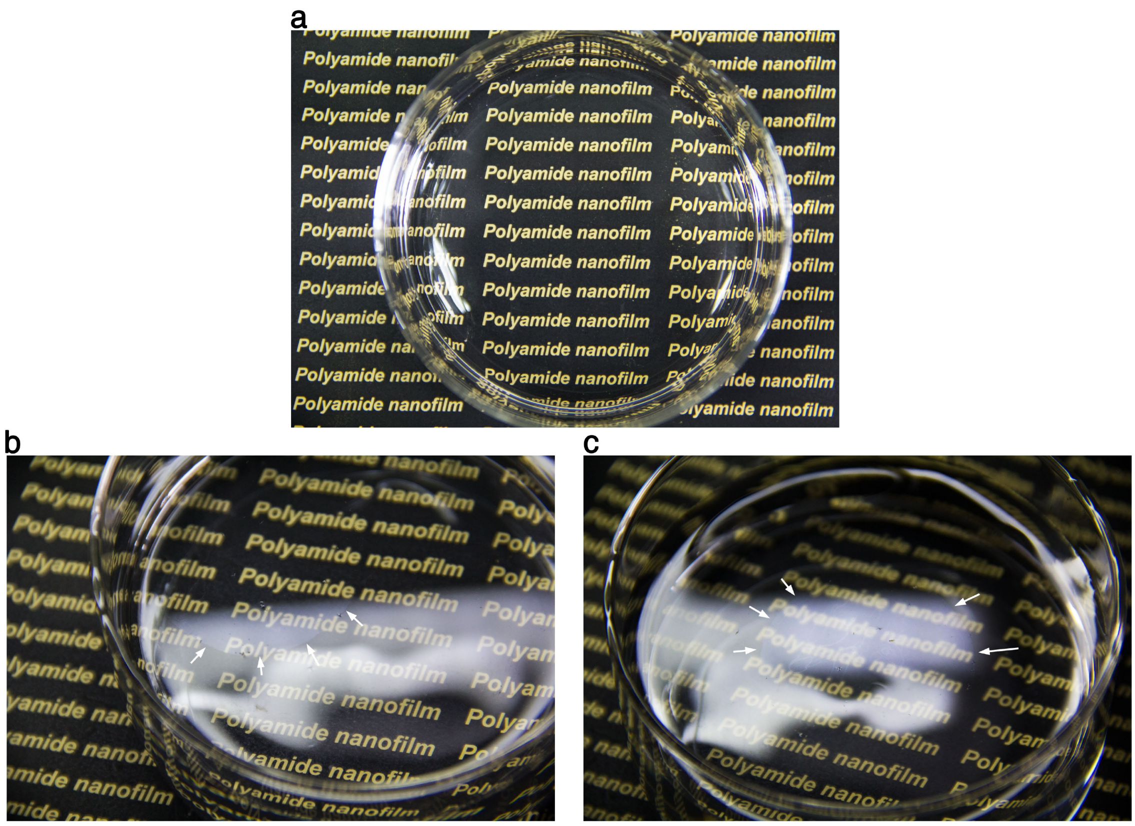

Figure S30. Photographs of an ultrathin free-standing polyamide nanofilm (sub 5 nm) floating on the water surface. The nanofilm is too thin to be observed from the front view a), but can be observed from the side view b) and c) (5 mM BAP reacted with 0.06 mM TMC, 15 min).

2.2.16. Gas separation

The gas permeance was measured by a bubble flowmeter with a total volume of 25 mL with 0.1 mL accuracy. The composite membrane was immobilized in an in-line stainless steel filter holder with an effective membrane surface area of 2.2 cm^2 (purchased from Millipore, US). The gas permeance measurements were performed more than three times for each sample, and three membranes were tested with errors within ±15%. Gas permeance (Q) was calculated by Equation S9:

$$Q = \frac{1}{P_u - P_d} \times \frac{273.15}{273.15+T} \times \frac{P_{atm}}{101.325} \times \frac{1}{A} \times \frac{dV}{dt} \qquad \text{(S9)}$$

where P_u is the upstream pressure, P_d is the downstream pressure (1 atm), P_{atm} is the atmospheric pressure, A is the effective membrane area, T is the temperature, dV/dt is the volumetric displacement rate in the bubble flow meter. The ideal selective factor of two gases is the ratio of two gas permeance (Equation S10):

$$\alpha = \frac{Q_1}{Q_2} \qquad \text{(S10)}$$

where Q_1 and Q_2 are the permeance of two gases, respectively. The gas permeability was calculated by multiplying permeance (Q) and thickness.

Table S10: Data points of H_2 and CO_2 permeability and selectivity in Figure 5g.

H_2 permeability (barrer)	CO_2 permeability (barrer)	H_2/CO_2 selectivity	Reference
40.2	9.2	4.37	[111]
106	40.1	2.64	
516	431	1.20	
45.5	15.3	2.97	
119	42.7	2.79	
549	440	1.25	
202	137	1.47	
70	30.9	2.27	
51	27	1.89	[112]
41	20	2.05	
100	63	1.59	
91	71	1.28	
110	67	1.64	
230	200	1.15	
41	22	1.86	

85	63	1.35	
130	110	1.18	
55	39	1.41	
150	95	1.58	
22	6.7	3.28	
77	54	1.43	
25	7.8	3.21	
43	19	2.26	
350	360	0.97	[113]
210	190	1.11	
100	62	1.61	
72	32	2.25	
257	189	1.36	[114]
36	24	1.50	[115]
207	73	2.84	
206	82	2.51	
248	126	1.97	
376	234	1.61	
44	11	4.00	
1779	1624	1.10	
1231	912	1.35	[116]
1062	759	1.40	
942	702	1.34	
470	325	1.45	
738	295	2.50	
30	5	6.00	
14.3	2.7	5.30	[117]
38.2	11.4	3.35	
47.1	25.3	1.86	
623	389	1.60	
1228	1014	1.21	
35	9.9	3.54	[118]
4194	4201	1.00	
1989	1874	1.06	
2895	1805	1.60	
1680	525	3.20	
5200	19000	0.27	[119]
300	560	0.54	
270	310	0.87	
180	150	1.20	

290	290	1.00	
140	130	1.08	
76	130	0.58	
76	170	0.45	
66	130	0.51	
100	180	0.56	
53	71	0.75	
84	120	0.70	
42	70	0.60	
43	25	1.72	
57	40	1.43	
45	48	0.94	
29	23	1.26	
39	15	2.60	
29	54	0.54	
3042	5366	0.57	[120]
3364	7329	0.46	
2347	4646	0.51	
1478	2627	0.56	[121]
3049	5799	0.53	
1368	3056	0.45	
2616	4756	0.55	
2854	5320	0.54	
3567	6441	0.55	
1703	3065	0.56	
2077	3693	0.56	
2695	4814	0.56	
3847	7108	0.54	
1300	2300	0.57	[122]
860	1100	0.78	
530	1100	0.48	[123]
220	210	1.05	
360	520	0.69	
300	420	0.71	
350	510	0.69	
1600	3700	0.43	
1020	2270	0.45	
600	1070	0.56	[124]
900	2000	0.45	
1100	2570	0.43	

1700	4600	0.37
2100	5400	0.39
2150	5300	0.41
1500	3800	0.39
1300	3400	0.38
2100	6100	0.34
3300	11200	0.29

References

[1] Y. Zhao, D. G. Truhlar. *Theor. Chem. Acc.* **2008**, *120*, 215-241.
[2] R. Krishnan, J. S. Binkley, R. Seeger, J. A. Pople. *J. Chem. Phys.* **1980**, *72*, 650-654.
[3] S. Grimme, J. Antony, S. Ehrlich, H. Krieg. *J. Chem. Phys.* **2010**, *132*, 154104.
[4] T. Lu, F. Chen. *J. Comput. Chem.* **2012**, *33*, 580-592.
[5] T. Lu, S. Manzetti. *Struct. Chem.* **2014**, *25*, 1521-1533.
[6] M. J. T. Frisch, G. W.; Schlegel, H. B.; Scuseria, G. E.; Robb, M. A.; Cheeseman, J. R.; Scalmani, G.; Barone, V.; Mennucci, B.; Petersson, G. A.; Nakatsuji, H.; Caricato, M.; Li, X.; Hratchian, H. P.; Izmaylov, A. F.; Bloino, J.; Zheng, G.; Sonnenberg, J. L.; Hada, M.; Ehara, M.; Toyota, K.; Fukuda, R.; Hasegawa, J.; Ishida, M.; Nakajima, T.; Honda, Y.; Kitao, O.; Nakai, H.; Vreven, T.; Montgomery, J. A., Jr.; Peralta, J. E.; Ogliaro, F.; Bearpark, M.; Heyd, J. J.; Brothers, E.; Kudin, K. N.; Staroverov, V. N.; Kobayashi, R.; Normand, J.; Raghavachari, K.; Rendell, A.; Burant, J. C.; Iyengar, S. S.; Tomasi, J.; Cossi, M.; Rega, N.; Millam, J. M.; Klene, M.; Knox, J. E.; Cross, J. B.; Bakken, V.; Adamo, C.; Jaramillo, J.; Gomperts, R.; Stratmann, R. E.; Yazyev, O.; Austin, A. J.; Cammi, R.; Pomelli, C.; Ochterski, J. W.; Martin, R. L.; Morokuma, K.; Zakrzewski, V. G.; Voth, G. A.; Salvador, P.; Dannenberg, J. J.; Dapprich, S.; Daniels, A. D.; Farkas, O.; Foresman, J. B.; Ortiz, J. V.; Cioslowski, J.; Fox, D. J. Gaussian 09, revision C.01; Gaussian Inc.: Wallingford, CT, 2016.
[7] S. V. Sambasivarao, O. Acevedo. *J. Chem. Theory Comput.* **2009**, *5*, 1038-1050.
[8] B. Doherty, X. Zhong, S. Gathiaka, B. Li, O. Acevedo. *J. Chem. Theory Comput.* **2017**, *13*, 6131-6145.
[9] W. L. Jorgensen, D. S. Maxwell, J. Tirado-Rives. *J. Am. Chem. Soc.* **1996**, *118*, 11225-11236.
[10] H. W. Horn, W. C. Swope, J. W. Pitera, J. D. Madura, T. J. Dick, G. L. Hura, T. Head-Gordon. *J. Chem. Phys.* **2004**, *120*, 9665-9678.
[11] T. Darden, D. York, L. Pedersen. *J. Chem. Phys.* **1993**, *98*, 10089-10092.
[12] L. Martínez, R. Andrade, E. G. Birgin, J. M. Martínez. *J. Comput. Chem.* **2009**, *30*, 2157-2164.
[13] M. C. Payne, M. P. Teter, D. C. Allan, T. A. Arias, J. D. Joannopoulos. *Rev. Mod. Phys.* **1992**, *64*, 1045-1097.
[14] G. Bussi, D. Donadio, M. Parrinello. *J. Chem. Phys.* **2007**, *126*, 014101.
[15] H. J. C. Berendsen, J. P. M. Postma, W. F. van Gunsteren, A. DiNola, J. R. Haak. *J. Chem. Phys.* **1984**, *81*, 3684-3690.
[16] M. J. Abraham, T. Murtola, R. Schulz, S. Páll, J. C. Smith, B. Hess, E. Lindahl. *SoftwareX* **2015**, *1-2*, 19-25.
[17] M. Luo, L. Dai. *J. Phys.: Condens. Matter* **2007**, *19*.
[18] A. Ghoufi, P. Malfreyt, D. J. Tildesley. *Chem. Soc. Rev.* **2016**, *45*, 1387-1409.
[19] M. Brehm, B. Kirchner. *J. Chem. Inf. Model.* **2011**, *51*, 2007-2023.
[20] W. Humphrey, A. Dalke, K. Schulten. *J. Mol. Graph.* **1996**, *14*, 33-38.
[21] B. Wang, L. Qin, T. Mu, Z. Xue, G. Gao. *Chem. Rev.* **2017**, *117*, 7113-7131.
[22] V. K. Aggarwal, I. Emme, A. Mereu. *Chem. Commun. (Camb.)* **2002**, 1612-1613.
[23] S. T. Handy, M. Okello. *The Journal of Organic Chemistry* **2005**, *70*, 1915-1918.
[24] L. C. Branco, J. G. Crespo, C. A. M. Afonso. *Angew. Chem. Int. Ed.* **2002**, *41*, 2771-2773.

[25] E. D. Bates, R. D. Mayton, I. Ntai, J. H. Davis. *J. Am. Chem. Soc.* **2002**, *124*, 926-927.
[26] A. V. Marenich, C. J. Cramer, D. G. Truhlar. *J. Phys. Chem. B* **2009**, *113*, 6378-6396.
[27] J. Ho, A. Klamt, M. L. Coote. *J. Phys. Chem. A* **2010**, *114*, 13442-13444.
[28] Z. Tan, S. Chen, X. Peng, L. Zhang, C. Gao. *Science* **2018**, *360*, 518.
[29] S. Gao, Y. Zhu, Y. Gong, Z. Wang, W. Fang, J. Jin. *ACS Nano* **2019**, *13*, 5278-5290.
[30] F.-Y. Zhao, Y.-L. Ji, X.-D. Weng, Y.-F. Mi, C.-C. Ye, Q.-F. An, C.-J. Gao. *ACS Appl. Mater. Interfaces* **2016**, *8*, 6693-6700.
[31] X. Zhu, H. Liang, X. Tang, L. Bai, X. Zhang, Z. Gan, X. Cheng, X. Luo, D. Xu, G. Li. *ACS Appl. Mater. Interfaces* **2019**, *11*, 21137-21149.
[32] G. Gong, P. Wang, Z. Zhou, Y. Hu. *ACS Appl. Mater. Interfaces* **2019**, *11*, 7349-7356.
[33] B. Yuan, C. Jiang, P. Li, H. Sun, P. Li, T. Yuan, H. Sun, Q. J. Niu. *ACS Appl. Mater. Interfaces* **2018**, *10*, 43057-43067.
[34] J.-J. Wang, H.-C. Yang, M.-B. Wu, X. Zhang, Z.-K. Xu. *J. Mater. Chem. A* **2017**, *5*, 16289-16295.
[35] R. Zhang, Y. Li, Y. Su, X. Zhao, Y. Liu, X. Fan, T. Ma, Z. Jiang. *J. Mater. Chem. A* **2016**, *4*, 7892-7902.
[36] Z. Zhai, C. Jiang, N. Zhao, W. Dong, H. Lan, M. Wang, Q. J. Niu. *J. Mater. Chem. A* **2018**, *6*, 21207-21215.
[37] J. Zhu, J. Hou, R. Zhang, S. Yuan, J. Li, M. Tian, P. Wang, Y. Zhang, A. Volodin, B. Van der Bruggen. *J. Mater. Chem. A* **2018**, *6*, 15701-15709.
[38] S. Liu, C. Wu, W.-S. Hung, X. Lu, K.-R. Lee. *J. Mater. Chem. A* **2017**, *5*, 22988-22996.
[39] Z. Wang, Z. Wang, S. Lin, H. Jin, S. Gao, Y. Zhu, J. Jin. *Nat. Commun.* **2018**, *9*, 2004.
[40] Y. Zhu, W. Xie, S. Gao, F. Zhang, W. Zhang, Z. Liu, J. Jin. *Small* **2016**, *12*, 5034-5041.
[41] H. Peng, Q. Tang, S. Tang, J. Gong, Q. Zhao. *J. Membr. Sci.* **2019**, *592*, 117386.
[42] M.-B. Wu, Y. Lv, H.-C. Yang, L.-F. Liu, X. Zhang, Z.-K. Xu. *J. Membr. Sci.* **2016**, *515*, 238-244.
[43] S. Huang, M.-B. Wu, C.-Y. Zhu, M.-Q. Ma, J. Yang, J. Wu, Z.-K. Xu. *ACS Sustain. Chem. Eng.* **2019**, *7*, 12315-12322.
[44] X. Zhang, C. Liu, J. Yang, C.-Y. Zhu, L. Zhang, Z.-K. Xu. *J. Membr. Sci.* **2020**, *593*, 117444.
[45] X. Zhang, Y. Lv, H.-C. Yang, Y. Du, Z.-K. Xu. *ACS Appl. Mater. Interfaces* **2016**, *8*, 32512-32519.
[46] K. Gu, S. Wang, Y. Li, X. Zhao, Y. Zhou, C. Gao. *J. Membr. Sci.* **2019**, *581*, 214-223.
[47] Z. Zhang, G. Kang, H. Yu, Y. Jin, Y. Cao. *J. Membr. Sci.* **2019**, *570-571*, 403-409.
[48] C. Jiang, L. Tian, Z. Zhai, Y. Shen, W. Dong, M. He, Y. Hou, Q. J. Niu. *J. Membr. Sci.* **2019**, *589*, 117244.
[49] J. Yuan, M. Wu, H. Wu, Y. Liu, X. You, R. Zhang, Y. Su, H. Yang, J. Shen, Z. Jiang. *J. Mater. Chem. A* **2019**, *7*, 25641-25649.
[50] M. B. M. Y. Ang, C. A. Trilles, M. R. De Guzman, J. M. Pereira, R. R. Aquino, S.-H. Huang, C.-C. Hu, K.-R. Lee, J.-Y. Lai. *Sep. Purif. Technol.* **2019**, *224*, 113-120.
[51] Y. Kang, M. Obaid, J. Jang, I. S. Kim. *Desalination* **2019**, *470*, 114125.
[52] X. Zhu, X. Cheng, J. Xing, T. Wang, D. Xu, L. Bai, X. Luo, W. Wang, G. Li, H. Liang. *Desalination* **2020**, *474*, 114197.
[53] S. Al Aani, A. Haroutounian, C. J. Wright, N. Hilal. *Desalination* **2018**, *427*, 60-74.
[54] S. Xiong, D. Y. Zhang, S. Mei, J. Liu, Y. S. Shi, Y. Wang. *J. Membr. Sci.* **2018**, *551*, 294-304.
[55] Z. Yang, Z.-w. Zhou, H. Guo, Z. Yao, X.-h. Ma, X. Song, S.-P. Feng, C. Y. Tang. *Environ. Sci. Technol.* **2018**, *52*, 9341-9349.
[56] Y. Li, E. Wong, Z. Mai, B. Van der Bruggen. *J. Membr. Sci.* **2019**, *592*, 117396.
[57] C. Wang, Z. Li, J. Chen, Z. Li, Y. Yin, L. Cao, Y. Zhong, H. Wu. *J. Membr. Sci.* **2017**, *523*, 273-281.
[58] J. Zheng, M. Li, K. Yu, J. Hu, X. Zhang, L. Wang. *J. Membr. Sci.* **2017**, *524*, 344-353.
[59] H. Zhang, L. Bin, J. Pan, Y. Qi, J. Shen, C. Gao, B. Van der Bruggen. *J. Membr. Sci.* **2017**, *539*, 128-137.
[60] G. S. Lai, W. J. Lau, P. S. Goh, A. F. Ismail, Y. H. Tan, C. Y. Chong, R. Krause-Rehberg, S. Awad. *Chem. Eng. J.* **2018**, *344*, 524-534.
[61] F. Soyekwo, Q. Zhang, R. Gao, Y. Qu, C. Lin, X. Huang, A. Zhu, Q. Liu. *J. Membr. Sci.* **2017**, *524*, 174-185.
[62] P. Wen, Y. Chen, X. Hu, B. Cheng, D. Liu, Y. Zhang, S. Nair. *J. Membr. Sci.* **2017**, *535*, 208-220.
[63] H.-Z. Zhang, Z.-L. Xu, H. Ding, Y.-J. Tang. *Desalination* **2017**, *420*, 158-166.

[64] Dupont, filmtec™ NF270 nanofiltration elements datasheet no. 45-d01529-en, rev. 3. https://www.dupont.com/content/dam/dupont/amer/us/en/water-solutions/public/documents/en/45-D01529-en.pdf, accessed: 11, **2020**.

[65] Suez water technologies & solutions, HL series water softening nf elements datasheet fs1273en.Docx jan-18. https://my.suezwatertechnologies.com/WTSCustomerPortal/s/content-download?DN=FS1273EN.pdf, accessed: 11, **2020**.

[66] Suez water technologies & solutions, HP series industrial low pressure nf elements datasheet fssmhpseries_en.Docx nov-18. https://my.suezwatertechnologies.com/WTSCustomerPortal/s/content-download?DN=FSsmHPSeries_EN.pdf, accessed: 11, **2020**.

[67] Suez water technologies & solutions, DK series industrial high rejection nanofiltration elements datasheet am-fspwdkseires_en.Docx nov-18. https://my.suezwatertechnologies.com/WTSCustomerPortal/s/content-download?DN=AM-FSpwDKSeires_EN.pdf, accessed: 11, **2020**.

[68] Suez water technologies & solutions, industrial NF1 series industrial high-pressure nanofiltration elements datasheet fssmindustrialnf1_en.Docx jan-18. https://my.suezwatertechnologies.com/WTSCustomerPortal/s/content-download?DN=FSpsDuraconNF1_EN.pdf, accessed: 11, **2020**.

[69] Toray csm membrane technologies, CSM brackish water membrane datasheet ne8040-hrm. http://www.csmfilter.com/csm/upload/RO_Catalogue/CSM%20RO%20catalogue.pdf, accessed: 11, **2020**.

[70] Hydranautics, NANO-SW-4040 datasheet 12/4/18. https://membranes.com/wp-content/uploads/Documents/Element-Specification-Sheets/NF/NANO-SW/NANO-SW-4040.pdf, accessed: 11, **2020**.

[71] M. R. Chowdhury, J. Steffes, B. D. Huey, J. R. McCutcheon. *Science* **2018**, *361*, 682.

[72] Z. Jiang, S. Karan, A. G. Livingston. *Adv. Mater.* **2018**, *30*, 1705973.

[73] J.-E. Gu, S. Lee, C. M. Stafford, J. S. Lee, W. Choi, B.-Y. Kim, K.-Y. Baek, E. P. Chan, J. Y. Chung, J. Bang, J.-H. Lee. *Adv. Mater.* **2013**, *25*, 4778-4782.

[74] W. Choi, J.-E. Gu, S.-H. Park, S. Kim, J. Bang, K.-Y. Baek, B. Park, J. S. Lee, E. P. Chan, J.-H. Lee. *ACS Nano* **2015**, *9*, 345-355.

[75] S.-J. Park, W.-G. Ahn, W. Choi, S.-H. Park, J. S. Lee, H. W. Jung, J.-H. Lee. *J. Mater. Chem. A* **2017**, *5*, 6648-6655.

[76] C. H. Park, S. Jeon, S.-H. Park, M. G. Shin, M. S. Park, S.-Y. Lee, J.-H. Lee. *J. Mater. Chem. A* **2019**, *7*, 3992-4001.

[77] J. Lee, J. H. Jang, H.-R. Chae, S. H. Lee, C.-H. Lee, P.-K. Park, Y.-J. Won, I.-C. Kim. *J. Mater. Chem. A* **2015**, *3*, 22053-22060.

[78] H. J. Kim, M.-Y. Lim, K. H. Jung, D.-G. Kim, J.-C. Lee. *J. Mater. Chem. A* **2015**, *3*, 6798-6809.

[79] X.-H. Ma, Z.-K. Yao, Z. Yang, H. Guo, Z.-L. Xu, C. Y. Tang, M. Elimelech. *Environ. Sci. Technol. Lett.* **2018**, *5*, 123-130.

[80] C. Liu, Y. Liu, Y. Guo, C. Wang, Z. Hu, C. Zhang. *Chem. Eng. J.* **2019**, *357*, 269-279.

[81] R. Yang, J. Xu, G. Ozaydin-Ince, S. Y. Wong, K. K. Gleason. *Chem. Mater.* **2011**, *23*, 1263-1272.

[82] M. Ben-Sasson, X. Lu, S. Nejati, H. Jaramillo, M. Elimelech. *Desalination* **2016**, *388*, 1-8.

[83] W. Choi, S. Jeon, S. J. Kwon, H. Park, Y.-I. Park, S.-E. Nam, P. S. Lee, J. S. Lee, J. Choi, S. Hong, E. P. Chan, J.-H. Lee. *J. Membr. Sci.* **2017**, *527*, 121-128.

[84] S. Liu, Z.-X. Low, H. M. Hegab, Z. Xie, R. Ou, G. Yang, G. P. Simon, X. Zhang, L. Zhang, H. Wang. *J. Membr. Sci.* **2019**, *592*, 117363.

[85] X. Song, B. Gan, Z. Yang, C. Y. Tang, C. Gao. *J. Membr. Sci.* **2019**, *582*, 342-349.

[86] X. Ma, Z. Yang, Z. Yao, H. Guo, Z. Xu, C. Y. Tang. *J. Colloid Interface Sci.* **2019**, *540*, 382-388.

[87] Z. Yang, X. Huang, X.-h. Ma, Z.-w. Zhou, H. Guo, Z. Yao, S.-P. Feng, C. Y. Tang. *J. Membr. Sci.* **2019**, *570-571*, 314-321.

[88] M. Fathizadeh, H. N. Tien, K. Khivantsev, Z. Song, F. Zhou, M. Yu. *Desalination* **2019**, *451*, 125-132.

[89] D. Ma, S. B. Peh, G. Han, S. B. Chen. *ACS Appl. Mater. Interfaces* **2017**, *9*, 7523-7534.

[90] Dupont, filmtec™ SW30XHR-440i element datasheet no. 45-d00968-en, rev. 5. https://www.dupont.com/content/dam/dupont/amer/us/en/water-solutions/public/documents/en/45-D00968-en.pdf, accessed: 11, **2020**.

[91] Dupont, filmtec™ BW30-400/34i element datasheet no. 45-d01528-en, rev. 5. https://www.dupont.com/content/dam/dupont/amer/us/en/water-solutions/public/documents/en/45-D01528-en.pdf, accessed: 11, **2020**.
[92] Suez water technologies & solutions, Duraslick RO series low fouling RO elements datasheet fssmduraslickro_en.Docx jul-18. https://my.suezwatertechnologies.com/WTSCustomerPortal/s/content-download?DN=FSsmDuraSlickRO_EN.pdf, accessed: 11, **2020**.
[93] Suez water technologies & solutions, Polisher RO permeate & condensate polishing datasheet fssmpolisherro_en.Docx jun-18. https://my.suezwatertechnologies.com/WTSCustomerPortal/s/content-download?DN=FSsmPolisherRO_EN.pdf, accessed: 11, **2020**.
[94] Suez water technologies & solutions, Industrial high pressure brackish water RO elements datasheet fssmindustrialroseries_en.Docx sep-19. https://my.suezwatertechnologies.com/WTSCustomerPortal/s/content-download?DN=FSsmIndustrialROSeries_EN.pdf, accessed: 11, **2020**.
[95] Suez water technologies & solutions, Muni Ro ULE series reverse osmosis ultra-low energy membrane elements for municipal drinking water plants datasheet https://my.suezwatertechnologies.com/WTSCustomerPortal/s/content-download?DN=FSsmMUNI_RO_ULE_EN.pdf, accessed: 11, **2020**.
[96] Hydranautics, SWC4-LD datasheet 1/30/19. https://membranes.com/wp-content/uploads/Documents/Element-Specification-Sheets/RO/SWC/SWC4-LD.pdf, accessed: 11, **2020**.
[97] Hydranautics, CPA2-4040 datasheet 12/4/18. https://membranes.com/wp-content/uploads/Documents/Element-Specification-Sheets/RO/CPA/CPA2-4040.pdf, accessed: 11, **2020**.
[98] Hydranautics, ESPA1-LD-4040 datasheet 12/4/18. https://membranes.com/wp-content/uploads/Documents/Element-Specification-Sheets/RO/ESPA/ESPA1-LD-4040.pdf, accessed: 11, **2020**.
[99] Toray csm membrane technologies, CSM brackish water membrane datasheet re4040-be. http://www.csmfilter.com/csm/upload/RO_Catalogue/CSM%20RO%20catalogue.pdf, accessed: 11, **2020**.
[100] H. J. Kim, K. Choi, Y. Baek, D.-G. Kim, J. Shim, J. Yoon, J.-C. Lee. *ACS Appl. Mater. Interfaces* **2014**, *6*, 2819-2829.
[101] L. J. Abbott, K. E. Hart, C. M. Colina. *Theor. Chem. Acc.* **2013**, *132*, 1334.
[102] T. F. Willems, C. H. Rycroft, M. Kazi, J. C. Meza, M. Haranczyk. *Microporous Mesoporous Mater.* **2012**, *149*, 134-141.
[103] M. Pinheiro, R. L. Martin, C. H. Rycroft, A. Jones, E. Iglesia, M. Haranczyk. *J. Mol. Graph. Model.* **2013**, *44*, 208-219.
[104] T. Lu, F. Chen. *J. Mol. Graph. Model.* **2012**, *38*, 314-323.
[105] L. F. Villalobos, T. Huang, K.-V. Peinemann. *Adv. Mater.* **2017**, *29*, 1606641.
[106] L. Huang, J. Chen, T. Gao, M. Zhang, Y. Li, L. Dai, L. Qu, G. Shi. *Adv. Mater.* **2016**, *28*, 8669-8674.
[107] S. Karan, S. Samitsu, X. Peng, K. Kurashima, I. Ichinose. *Science* **2012**, *335*, 444.
[108] S. Karan, Z. Jiang, A. G. Livingston. *Science* **2015**, *348*, 1347.
[109] M. F. Jimenez-Solomon, Q. Song, K. E. Jelfs, M. Munoz-Ibanez, A. G. Livingston. *Nat. Mater.* **2016**, *15*, 760-767.
[110] T. Huang, T. Puspasari, S. P. Nunes, K.-V. Peinemann. *Adv. Funct. Mater.* **2020**, *30*, 1906797.
[111] K. Tanaka, M. Okano, H. Toshino, H. Kita, K.-I. Okamoto. *J. Polym. Sci. Pol. Phys.* **1992**, *30*, 907-914.
[112] M. Al-Masri, D. Fritsch, H. R. Kricheldorf. *Macromolecules* **2000**, *33*, 7127-7135.
[113] M. Al-Masri, H. R. Kricheldorf, D. Fritsch. *Macromolecules* **1999**, *32*, 7853-7858.
[114] Y. J. Cho, H. B. Park. *Macromol. Rapid Commun.* **2011**, *32*, 579-586.
[115] S. H. Han, J. E. Lee, K.-J. Lee, H. B. Park, Y. M. Lee. *J. Membr. Sci.* **2010**, *357*, 143-151.
[116] H. B. Park, S. H. Han, C. H. Jung, Y. M. Lee, A. J. Hill. *J. Membr. Sci.* **2010**, *359*, 11-24.
[117] C. H. Jung, J. E. Lee, S. H. Han, H. B. Park, Y. M. Lee. *J. Membr. Sci.* **2010**, *350*, 301-309.
[118] J. I. Choi, C. H. Jung, S. H. Han, H. B. Park, Y. M. Lee. *J. Membr. Sci.* **2010**, *349*, 358-368.

[119] S. Alexander Stern. *J. Membr. Sci.* **1994**, *94*, 1-65.
[120] N. Du, G. P. Robertson, I. Pinnau, S. Thomas, M. D. Guiver. *Macromol. Rapid Commun.* **2009**, *30*, 584-588.
[121] N. Du, G. P. Robertson, I. Pinnau, M. D. Guiver. *Macromolecules* **2010**, *43*, 8580-8587.
[122] P. M. Budd, K. J. Msayib, C. E. Tattershall, B. S. Ghanem, K. J. Reynolds, N. B. McKeown, D. Fritsch. *J. Membr. Sci.* **2005**, *251*, 263-269.
[123] B. S. Ghanem, N. B. McKeown, P. M. Budd, N. M. Al-Harbi, D. Fritsch, K. Heinrich, L. Starannikova, A. Tokarev, Y. Yampolskii. *Macromolecules* **2009**, *42*, 7881-7888.
[124] D. Fritsch, G. Bengtson, M. Carta, N. B. McKeown. *Macromol. Chem. Phys.* **2011**, *212*, 1137-1146.

Author Contributions

C.L., J.Y., and Z.K.X conceived the concept and designed experiments. C.L. performed the experiments and computer simulations. C.L., J.Y., B.B.G and Z.K.X analyzed the data and discussed the results. C.L., J.Y., S.A., A.G and Z.K.X wrote the manuscript. C.L. and J.Y. contributed equally to this work.
